# Cyclic nucleotide‐gated channels 14, 15, and 16 coordinate multiple signaling pathways to promote salt tolerance in rice

**DOI:** 10.1111/tpj.71092

**Published:** 2026-08-19

**Authors:** Lilin Luo, Xiaoli Gong, Dongyang Wang, Yanhua Chen, Baohong Zou, Shan Lu

**Affiliations:** ^1^ State Key Laboratory of Crop Genetics & Germplasm Enhancement and Utilization, College of Agriculture Nanjing Agricultural University Nanjing 210095 China

**Keywords:** rice, Ca^2+^, ABA, ROS, CNGC, salt stress, stomata

## Abstract

Plants activate osmotic regulation, ion and reactive oxygen species (ROS) homeostasis modulation, and plant hormone abscisic acid (ABA)‐mediated stomatal movement regulation for survival under salt stress. However, it is unknown if and how a single molecule coordinates multiple responses. Here we characterized the functions of three closest homologs, *cyclic nucleotide‐gated channel 14*, *15*, and *16* (*OsCNGC14/15/16*), in regulating multiple signaling pathways in response to salt. Mutants of each of these three genes had reduced salt tolerance compared to the wild type. These mutants also had slower stomatal closure, lower Ca^2+^ influx, higher Na^+^/K^+^ ratio, and ROS accumulation under salt treatment. Conversely, overexpression of *OsCNGC16* led to enhanced salt tolerance, increased Ca^2+^ influx, lower Na^+^/K^+^ ratio, and ROS accumulation in response to salt. *OsCNGC14/15/16* also positively regulate shoot growth under ABA treatment. Exogenous ABA treatment rescued the defects in salt tolerance and stomatal closure of the *OsCNGC16* mutant. And treatment of ROS scavenger dimethylthiourea (DMTU) partially rescued the salt susceptibility of the *OsCNGC16* mutant. In addition, OsCNGC16 can interact with and be phosphorylated by Ca^2+^‐dependent protein kinases 5 and 13 (CPK5/13). This study unveils multiple roles of OsCNGC14/15/16 proteins in salt tolerance and identifies common regulators of stomatal closure, Ca^2+^ influx, Na^+^/K^+^ homeostasis, ROS scavenging, and ABA signaling in response to salt stress.

## Significance Statement

This study identifies cyclic nucleotide‐gated channel 14, 15, and 16 as positive regulators of salt tolerance in rice, and highlights the importance of *CNGC* genes in salt stress responses. They act as common regulators to coordinate stomatal closure, Ca^2+^ influx, Na^+^/K^+^ homeostasis, ROS scavenging, and ABA signaling pathways in response to salt stress.

## INTRODUCTION

Soil salinity is a great threat that limits plant growth, development, and crop yields (Patwa et al., 2025; Tariq et al., 2025; Tibesigwa et al., 2025). Root is the frontline organ experiencing salt stress (Cui et al., 2025). Salt stress reduces root mass and growth rate of the primary and lateral roots, thereby impairing plant growth (Cui et al., 2025). Salt stress can also affect aboveground tissues, leading to water deficit, and leaf wilting (Arif et al., 2020). Plants have evolved various adaptive mechanisms in response to the detrimental effects of salt stress, including regulation of ion homeostasis and cytoskeleton dynamics, osmotic adjustment to maintain cell turgor and water uptake, production of reactive oxygen species (ROS), and activation of calcium (Ca^2+^) and hormone signaling pathways (Zhao, Zhang, Liu, et al., 2021). Rice (*Oryza sativa*) is a salt‐sensitive crop which is exposed to salt stress at all developmental stages (Ganie et al., 2019; Qin et al., 2020). So, understanding the molecular mechanisms of salt tolerance regulation will provide efficient strategies for breeding salt‐adapted rice varieties.

Abscisic acid (ABA) is an important hormone that regulates stomatal closure and response to salt stress in plants (Chen et al., 2020; Zhu, 2002). The core of the ABA signaling network comprises a subfamily of type 2C protein phosphatases (PP2Cs) and three sucrose nonfermenting 1‐related kinases (SnRK2.2/2.3/2.6), and their activity are tightly controlled by ABA (Fujii et al., 2009; Umezawa et al., 2009). ABA can strongly activate kinase activity of SnRK2.2/2.3/2.6 (Boudsocq et al., 2004). In the absence of ABA, PP2Cs bind with SnRK2.2/2.3/2.6 to keep the kinases inactive by blocking their catalytic cleft and dephosphorylating the activation loop (Soon et al., 2012). In Arabidopsis, the *snrk2.2/2.3/2.6* triple mutant is extremely insensitive to ABA in stomatal closure (Fujii & Zhu, 2009). ABA promotes salt‐induced stomatal closure to reduce water loss and acts as the main modulator of salt stress response in roots (Geng et al., 2013; Niu et al., 2018; Zhao, Zhang, Teixeira da Silva, et al., 2021). Some ABA‐related genes are induced in roots under salt stress and regulate salt tolerance, such as *9‐cis‐epoxycarotenoid dioxygenase 4* (*OsNCED4*), *OsNCED5* and *ABA insensitive 5* (*OsABI5*; Huang et al., 2019; Li et al., 2022; Xiang et al., 2024). Although ABA has been proposed to be transported from roots to guard cells, stomatal closure is not solely dependent on ABA production in roots (Holbrook et al., 2002). So, ABA‐mediated salt stress responses entail complex plant signalings, and it is important to characterization new components involved in this process.

ROS act as early signaling molecules in response to salt stress and positively regulate salt tolerance through activating ABA‐dependent defense pathways (Ma et al., 2012). Nicotinamide adenine dinucleotide phosphate (NADPH) oxidase respiratory burst oxidase homolog (RBOH) is one of the key enzymes to generate ROS (Liu et al., 2020; Sun et al., 2025). In Arabidopsis, *AtRBOHD* and *AtRBOHF* promote ROS generation and positively regulate salt tolerance (Ma et al., 2012). The *JUN activation domain binding protein 1* (*OsJAB1*) negatively regulates salt tolerance by inhibiting early‐stage salt‐induced ROS accumulation (Wang, Zhang, et al., 2023). In rice, the *MADS‐box transcription factor 25* (*OsMADS25*), *OsMADS57*, and *OsWRKY70* positively regulate salt tolerance by maintaining ROS homeostasis (Ding et al., 2025; Sharma et al., 2014; Wu et al., 2021). On the other hand, rapid and excessive accumulation of ROS leads to toxic oxidative damage in plants (Yang & Guo, 2018). ROS‐scavenging enzymes can maintain ROS at appropriate levels to avoid toxicity, including ascorbate peroxidase (APX), superoxide dismutase (SOD), catalase (CAT), and glutathione peroxidase (GPX; Waszczak et al., 2018). Ascorbic acid (AsA) and glutathione (GSH) act as important non‐enzyme antioxidants to remove ROS, and the AsA‐GSH cycle is an important antioxidant defense system in response to salt stress (Lu et al., 2025; Prajapati et al., 2023). Although numerous molecular components have been reported to regulate salt tolerance through the modulation of ROS homeostasis in plants, the underlying mechanisms need to be further elucidated.

Plants have evolved sophisticated regulatory mechanisms to maintain ion (Na^+^, K^+^, and H^+^) homeostasis in response to salt stress (Cheema et al., 2025; Guo et al., 2023; Ma, Li, et al., 2025). The *high‐affinity potassium transporter* (*HKT*) genes are key ions transporters regulating Na^+^/K^+^ homeostasis in response to salt stress (Farooq et al., 2021). The high‐affinity K^+^ transporter (OsHAK1) positively maintains K^+^ ion homeostasis to promote salt tolerance in rice (Chen et al., 2015). In Arabidopsis, the salt overly sensitive (SOS) pathway alleviates salt stress by exporting excess Na^+^ (Shi et al., 2002). AtSOS2 phosphorylates AtSOS1 and enhances salt tolerance by increasing the Na^+^/H^+^ antiport activity (Van Zelm et al., 2020). Salt stress can also induce rise of the cytosolic free Ca^2+^ concentration and initial localized Ca^2+^ increases in roots immediately triggers systemic molecular responses in the whole plant (Choi et al., 2014; Yang et al., 2019, Ma, Li, et al., 2025). Changes in Ca^2+^ concentration under salt stress are always associated with the activation of Ca^2+^ channels. In rice, *Ca* ^*2+*^ *osmotic sensor reduced hyperosmolality‐induced Ca* ^*2+*^ *increase 1.1* (*OsOSCA1.1*) positively regulates salt‐induced Ca^2+^ influx and confers salt tolerance (Han et al., 2022). In Arabidopsis, Ca^2+^ influx channels *glutamate receptor‐like 3.4* (*AtGLR3.4*), *AtGLR3.6* and *AtGLR3.7* positively regulate salt tolerance (Cheng et al., 2018; Silamparasan et al., 2023; Wang et al., 2019). Some Ca^2+^ efflux pumps are also demonstrated to be responsible for Ca^2+^ homeostasis and salt tolerance, such as *autoinhibited Ca* ^*2+*^ *‐ATPase 2* (*AtACA2*) and *AtACA4* (Anil et al., 2008; Geisler et al., 2001).

As a group of Ca^2+^‐permeable nonspecific cation channels, cyclic nucleotide‐gated channels (CNGCs) have been reported to be involved in response to diverse abiotic stresses through regulating Ca^2+^ influx (Dietrich et al., 2020; Jarratt‐Barnham et al., 2021). In Arabidopsis, *AtCNGC20* positively regulates cold‐induced Ca^2+^ influx and confers freeing tolerance (Peng et al., 2024). *AtCNGC2* negatively regulates both basal and acquired heat tolerance at seedling stage (Finka et al., 2012; Lu, Zhu, et al., 2022). *AtCNGC5*, *6*, *9*, and *12* positively regulate ABA‐induced Ca^2+^ influx in guard cells and stomatal closure (Tan et al., 2022). In rice, *OsCNGC14*, *15*, and *16* (*OsCNGC14/15/16*) positively regulate Ca^2+^ influx and stomatal closure in response to heat, chilling, and the stress hormone ABA, which also enhance tolerance to heat, chilling, and drought (Cui et al., 2020; Luo et al., 2025). Only a few *CNGC* genes are reported to be involved in salt stress. In Arabidopsis, the expression of *AtCNGC19* and *AtCNGC20* is induced in the stems under salt stress (Kugler et al., 2009). *AtCNGC10* negatively regulates salt tolerance (Guo et al., 2010). Silencing of the *GhCNGC32* and *GhCNGC35* genes decreases salt tolerance in cotton (Lu, Yin, et al., 2022). Overexpression of the *GsCNGC20‐d* gene enhances salt tolerance in *Glycine soja* (Pi et al., 2023). *HcCNGC21* enhances activities of ROS‐scavenging enzymes (SOD, POD, and CAT) and positively regulates salt tolerance in kenaf (Chen, Wu, et al., 2024). OsCPK4 phosphorylates and activates OsCNGC7, thereby enhancing Ca^2+^ influx and salt stress tolerance in rice (Zhan et al., 2026). However, the function of *CNGCs* in regulating salt tolerance has not been fully elucidated in rice, and the mechanism of *CNGCs* in salt tolerance regulation needs to be further investigated in plants.

Here, we characterized the function of *OsCNGC14/15/16* in response to salt stress. We show that *OsCNGC14/15/16* are positive regulators of salt tolerance. They promote stomatal closure, Ca^2+^ influx, Na^+^/K^+^ homeostasis, and ROS scavenging in response to salt. Additionally, *OsCNGC14/15/16* positively regulate shoot growth under ABA treatment and exogenous ABA can rescue the defects in salt tolerance and stomatal closure of the *OsCNGC16* mutant. Furthermore, OsCNGC16 can directly interact with and be phosphorylated by the positive modulators of salt tolerance Ca^2+^‐dependent protein kinases 5 and 13 (CPK5/13). These studies reveal that CNGC proteins are co‐regulators in controlling stomatal movement, Ca^2+^ influx, Na^+^/K^+^ and ROS homeostasis, and ABA signaling to enhance salt tolerance.

## RESULTS

### *OsCNGC14/15/16* promote salt tolerance

**Figure 1:**
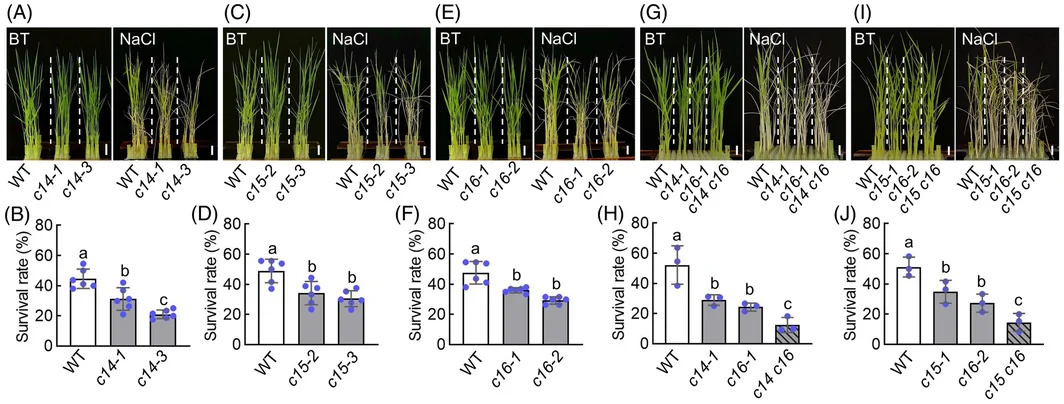
Salt tolerance of the *cngc14/15/16* single and double mutants. (A–J) Morphological phenotypes (A, C, E, G, I) and survival rates (B, D, F, H, J) of *cngc14* (*c14*; A, B), *cngc15* (*c15*; C, D), *cngc16* (*c16*; E, F), *cngc14 cngc16* (*c14 c16*; G, H), and *cngc15 cngc16* (*c15 16*; I, J) seedlings after treatment of 130 mM NaCl for 7 days compared with the wild type (WT). Photographs were taken before treatment (BT) and after recovery. Scale bars, 2 cm. Shown in (B, D, F, H, J) are means ± standard deviation (SD) from at least three biological replicates. Letters indicate significant differences at *P* < 0.01 by Duncan's multiple range test via ANOVA.

To investigate whether *OsCNGC14/15/16* are involved in salt stress response, we firstly examined their expression patterns in 2‐week‐old rice seedlings treated with 130 mM NaCl. Quantitative real‐time PCR (qRT‐PCR) revealed that all three genes exhibited slightly higher expression levels after 3 h of salt treatment compared with the untreated control (Figure S1a–c). We further used transgenic plants that contained the *β‐Glucuronidase* (*GUS*) reporter gene driven by the promoter of *OsCNGC16* to analyze whether or not *OsCNCG16* is salt‐induced. GUS staining signals were detected in the leaves and roots of 10‐days‐old seedlings, and the staining increased significantly after 24 h treatment of 130 mM NaCl (Figure S1d–f). The role of *OsCNGC14/15/16* in salt tolerance was assessed via their mutants previously generated by the CRISPR/*Cas9* gene editing system (Cui et al., 2020; Luo et al., 2025). After 130 mM NaCl treatment of 7 days followed by recovery growth, the *cngc14*, *cngc15*, and *cngc16* mutants had survival rates of 21–36%, lower than the rate of 44–45% for the wild type (WT; Figure 1A–F). Complementation transgenic lines for each of the three genes all had restored the salt tolerance levels comparable to those of the WT (Figure S2). Additionally, survival rates of two different *OsCNGC16* overexpression transgenic lines (OE‐*OsCNGC16*) were significantly higher than the WT (Figure S3). These results indicate that *OsCNGC14/15/16* are positive regulators of salt tolerance in rice. To explore the functional relationship among the three *OsCNGC* genes in salt tolerance, phenotypes of the double mutants were compared with the single mutants under 130 mM NaCl treatment. After salt treatment followed by recovery growth, the *cngc14 cngc16* and *cngc15 cngc16* double mutants had survival rates of 12–15%, lower than the 24–35% for the single mutants and 51–52% for the WT (Figure 1G–J). So, the double mutants of the three *OsCNGC* genes are more susceptible than each of the single mutants under salt treatment.

We also measured the shoot and root length of the *cngc* mutants and OE‐*OsCNGC16* plants in response to salt. A decrease in the shoot and root length was observed after 120 mM NaCl treatment, but there was no significant difference between the *cngc* mutants and the WT plants (Figure S4). Overexpression of *OsCNGC16* had no effect on the shoot length before and after 120 mM NaCl treatment (Figure S6a,b). However, OE‐*OsCNGC16*#1 and #2 had a significantly reduced root length compared to WT before and after treatment (Figure S6a,c). Further genotype by environment (G × E) interaction assays between each genotype pair (WT versus OE‐*OsCNGC16*) and salt treatment (CK, non‐treatment control versus NaCl) on root length showed that a significant interaction was found for the OE‐*OsCNGC16* genotype and salt treatment (Figure S6c), indicating that the inhibition by salt treatment on root length was weaker in OE‐*OsCNGC16* lines than in WT. Therefore, *OsCNGC14/1516* positively regulate salt tolerance of rice seedlings but may not contribute to seedling growth under salt stress.

### *OsCNGC14/15/16* promote stomatal closure and cytosolic Ca^2+^ influx in response to salt stress

**Figure 2:**
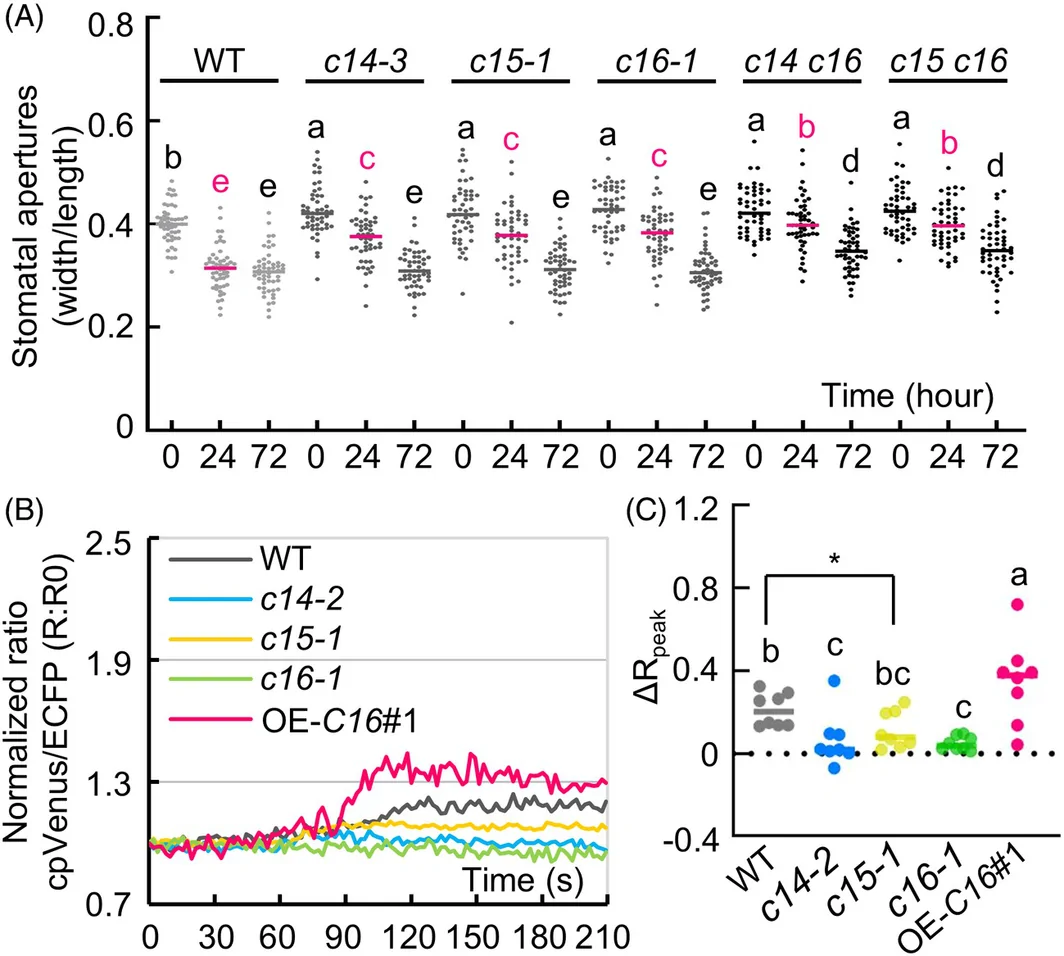
Stomatal movement and cytosolic Ca^2+^ influx in the *c14/15/16* mutants and OE‐*OsCNGC16* plants under salt stress. (A) Stomatal apertures of the WT, *c14‐3*, *c15‐1*, *c16‐1*, *c14 c16*, and *c15 c16* plants before and after treatment of 130 mM NaCl. Shown are dot plots from 50 stomata for each genotype. Letters indicate significant differences at *P* < 0.05 by Duncan's multiple range test via ANOVA. Pink highlights the mean value and the significance level when aperture reaches minimum in WT after treatment for each genotype. (B, C) Cytosolic Ca^2+^ signals measured by the YC3.6 reporter in WT, *c14‐2*, *c15‐1*, *c16‐1*, and OE‐*OsCNGC16*#1 (OE‐*C16*#1) plants before and after 130 mM NaCl treatment. Treatment was applied at 60 s from the start of the monitoring. The cytosolic Ca^2+^ levels were calculated by the cpVenus/ECFP ratio (R) and normalized by ratio at time point zero (R0). (B) are aggregated traces of Ca^2+^ levels expressed as R:R0 (showing mean values at each time point) from all eight monitored root cells (one cell/plant) for each genotype. (C) are dot plots of peak values Ca^2+^ influx ∆R_peak_ measured by ∆R that is the difference between R:R0 at the peak or highest measured (WT in 124 s, *cngcs* in 102 s, OE‐*C16*#1 in 120 s) and R:R0 right before stimulus is applied (60 s). Letters indicate significant differences at *P* < 0.05 by Duncan's multiple range test via ANOVA. * indicate significant differences compared to WT at *P* < 0.05 by two‐sided *t*‐test.

Given that *OsCNGC14/15/16* play important roles in salt tolerance and they promote stomatal closure in responses to heat, chilling, and drought (Luo et al., 2025), we characterized their roles in stomatal movement under salt stress. Under 130 mM NaCl treatment, the *cngc14‐3*, *cngc15‐1*, and *cngc16‐1* mutants exhibited delayed stomatal closure and attained their complete closure at 72 h, while the WT reached its aperture minimum at 24 h (Figure 2A). The *cngc14 cngc16* and *cngc15 cngc16* double mutants had larger stomata apertures compared to the *cngc* single mutants at 24 h and did not attain their complete closure at 72 h after salt treatment (Figure 2A). So, *OsCNGC14/15/16* positively regulate salt‐induced stomatal closure.

As Ca^2+^ channels, we further investigated whether or not *OsCNGC14/15/16* affect Ca^2+^ influx in the primary tissue of roots that experience salt stress (Jin et al., 2015; Munns & Tester, 2008). We analyzed Ca^2+^ influx in roots upon salt treatment in *cngc14‐2*, *cngc15‐1*, *cngc16‐1*, and OE‐*OsCNGC16*#1 carrying the Ca^2+^ reporter gene *yellow cameleon 3.6* (*YC3.6*; Krebs et al., 2012). In response to 130 mM NaCl treatment, WT root tip showed a significant cytosolic Ca^2+^ influx after salt treatment and maintained a high level of cytosolic Ca^2+^ during salt treatment, whereas reduced Ca^2+^ influx was observed in the three *cngc* mutants (Figure 2B). However, the cytosolic Ca^2+^ rose to a higher level in the OE‐*OsCNGC16*#1 plants in root tips compared to the WT (Figure 2B). Quantification of maximum increase of the FRET signal supports a reduction and an induction of Ca^2+^ influx in the three *cngc* and OE‐*OsCNGC16*#1 compared to the WT in response to salt stress, respectively (Figure 2C). These results indicate that *OsCNGC14/15/16* are essential for Ca^2+^ influx induced by salt in rice.

### *OsCNGC16* maintains Na^+^/K^+^ homeostasis under salt stress

**Figure 3:**
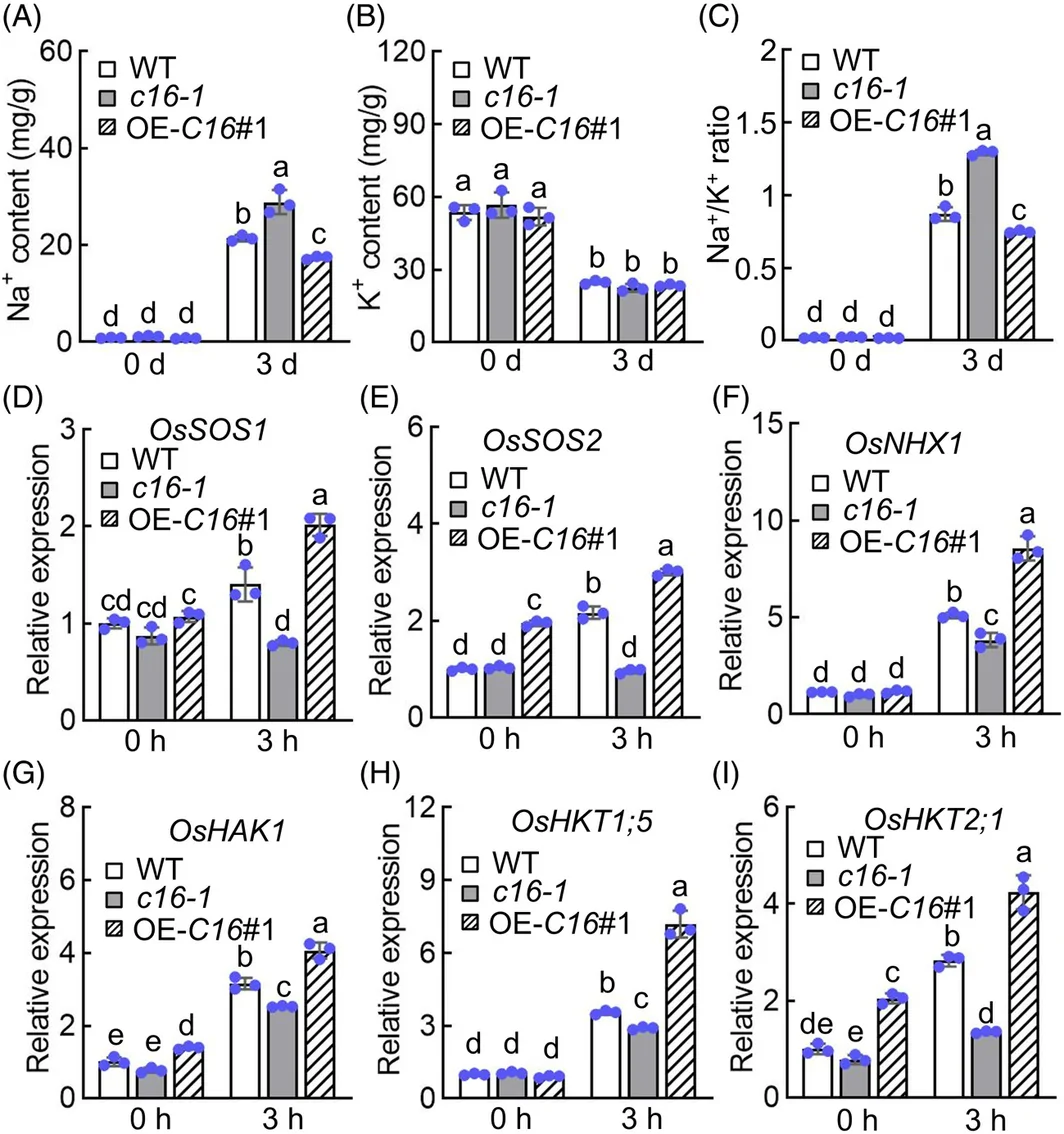
The Na^+^/K^+^ ratio and expression of Na^+^/K^+^ transporter genes in the *cngc16* and OE‐*OsCNGC16* plants under salt stress. (A–C) Na^+^ content (A), K^+^ content (B), and Na^+^/K^+^ ratio (C) in shoots of the WT, *c16‐1*, and OE‐*C16*#1 plants before and after treatment of 130 mM NaCl. (D–I) Relative expression levels of Na^+^/K^+^ transporter genes in the leaf tissues of 2‐week‐old WT, *c16‐1*, and OE‐*C16*#1 seedlings exposed to 130 mM NaCl for 0 and 3 h. Transcript levels were measured by quantitative real‐time PCR (qRT‐PCR) and represented as relative to the level of 0 h in WT. *OsActin1* was used as the reference gene. Shown are the means ± SD from three biological replicates. Letters indicate significant differences at *P* < 0.01 by Duncan's multiple range test via ANOVA.

The above results showed that *OsCNGC14/15/16* have similar functions based on their single mutant phenotypes under salt stress, and the *OsCNGC16* gene appears to have a larger role compared to the other two genes as the *cngc16‐1* mutants exhibited a stronger defect in Ca^2+^ influx under salt treatment (Figure 2B). So, we further investigated the mechanisms underlying the promotion of salt tolerance by *OsCNGC16*. The role of *OsCNGC16* in regulating Na^+^/K^+^ homeostasis under salt stress was analyzed through measuring Na^+^ and K^+^ contents in shoots of the *cngc16‐1* and OE‐*OsCNGC16*#1 plants. No significant differences in Na^+^ and K^+^ contents were observed among the WT, *cngc16‐1*, and OE‐*OsCNGC16*#1 plants before NaCl treatment (Figure 3A,B). After 3 days of 130 mM NaCl treatment, Na^+^ content increased in shoots of all the genotypes, and was significantly higher in *cngc16‐1* but lower in OE‐*OsCNGC16*#1 compared to WT (Figure 3A). K^+^ content decreased in shoots after salt treatment, and there was no significant difference among these genotypes (Figure 3B). The Na^+^/K^+^ ratio increased in shoots of all the genotypes, and was significantly higher in *cngc16‐1* but lower in OE‐*OsCNGC16*#1 compared to WT after salt treatment (Figure 3C). We further analyzed the expression of Na^+^/K^+^ transporter genes (Chen et al., 2015; Kobayashi et al., 2017; Kumar et al., 2022; Martínez‐Atienza et al., 2007; Theerawitaya et al., 2020) in *OsCNGC16* transgenic and WT plants before and after salt treatment. Under normal conditions, *OsSOS2*, *OsHAK1*, and *OsHKT2;1* had an evident increased expression in the OE‐*OsCNGC16*#1 plants compared with the WT (Figure 3E,G,I). After 3 h of 130 mM NaCl treatment, all of these genes (*OsSOS1*, *OsSOS2*, *OsNHX1*, *OsHAK1*, *OsHKT1;5* and *OsHKT2;1*) had a decreased expression in the *cngc16* mutant and an increased expression in the OE‐*OsCNGC16*#1 plants compared with the WT (Figure 3D–I). Taken together, *OsCNGC16* plays a critical role in maintaining Na^+^/K^+^ homeostasis in response to salt stress.

### Exogenous ABA contributes to *OsCNGC16* promoted salt tolerance

**Figure 4:**
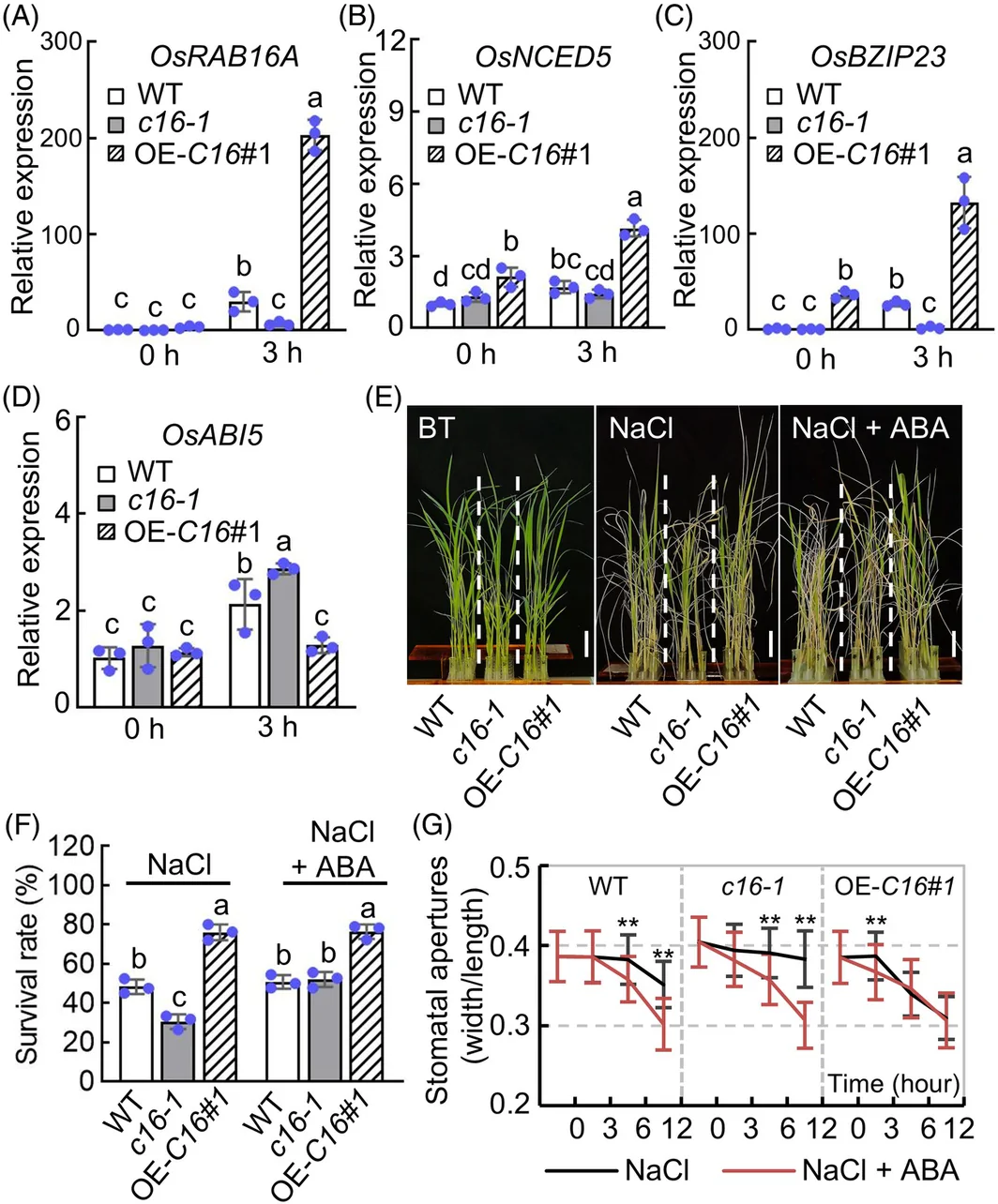
Effects of exogenous ABA salt tolerance and stomatal movement of the *cngc16* and OE‐*C16* plants. (A–D) Relative expression levels of ABA‐related genes in the leaf tissues of 2‐week‐old WT, *c16‐1*, and OE‐*C16*#1 seedlings exposed to 130 mM NaCl for 0 and 3 h. Transcript levels were measured by qRT‐PCR and represented as relative to the level of 0 h in WT. (E–G) Morphological phenotypes (E), survival rates (F), and quantification of stomatal apertures (G) of the WT, *c16‐1*, and OE‐*C16*#1 plants after treatment of 130 mM NaCl for 7 days with or without 100 μM ABA. Photographs were taken BT and after recovery. Scale bars, 2 cm. Shown in (F) are the means ± SD from at least three biological replicates. Shown in (G) are the means ± SD from 50 stomata for each genotype. Letters in (A–D, F) indicate significant differences compared to WT at *P* < 0.05 by Duncan's multiple range test via ANOVA. * or ** indicates significant differences compared to WT at *P* < 0.05 or 0.01 by two‐sided *t*‐test, respectively.

ABA‐mediated stomatal closure is crucial for salt tolerance in plants and *OsCNGC14/15/16* are essential for ABA‐induced stomatal closure (Bharath et al., 2021; Luo et al., 2025), suggesting that *OsCNGC14/15/16* may be involved in the regulation of salt tolerance through ABA signaling. To verify this, we firstly analyzed the growth phenotypes of the *cngc* mutants and overexpression transgenic lines under ABA treatment. A decrease in the shoot and root length was observed in all genotypes after ABA treatment (Figures S5 and S6). Under both 1 μM and 2 μM ABA treatment, shoot length of the *cngc* mutants was significantly shorter than the WT (Figure S5). No significant differences in root length were observed between the *cngc* mutants and WT before or after ABA treatment (Figure S5). Overexpression of *OsCNGC16* had no effect on the shoot length before and after ABA treatment (Figure S6a,b), but shorter roots in OE‐*OsCNGC16* #1 and #2 under normal conditions could not be observed after ABA treatment (Figure S6a,c). The expression of ABA‐related genes (Ganguly et al., 2012; Huang et al., 2019; Li et al., 2022; Xiang et al., 2008) was further analyzed by qRT‐PCR in the *OsCNGC16* transgenic and WT plants before and after salt treatment. Under normal conditions, *OsNCED5* and *OsBZIP23* had increased expression in the OE‐*OsCNGC16*#1 plants compared with the WT (Figure 4B,C). After 3 h of 130 mM NaCl treatment, *OsRAB16A* and *OsBZIP23* had decreased expression in the *cngc16‐1* mutant, while *OsRAB16A*, *OsNCED5*, and *OsBZIP23* had increased expression in the OE‐*OsCNGC16*#1 plants compared with the WT (Figure 4A–C). As negative regulator of salt tolerance, *OsABI5* had increased expression in the *cngc16‐1* mutant and decreased expression in the OE‐*OsCNGC16*#1 plants compared with the WT after salt treatment (Figure 4D).

We further analyzed the effect of exogenous ABA on salt tolerance of the *cngc16‐1* mutant and OE‐*OsCNGC16*#1 plants. Rice seedlings were pretreated with 100 μM ABA solution by spraying and followed by a salt treatment of 130 mM NaCl. After a recovery growth, the *cngc16‐1* mutant pretreated with ABA had a survival rate of 52%, the same level as WT, which was higher than the 30% for non‐pretreatment (Figure 4E,F). Measurement of stomatal aperture showed that ABA treatment could promote stomatal closure of the *cngc16‐1* mutant to the level of WT at 12 h under 130 mM NaCl treatment (Figure 4G). These results indicate that exogenous ABA can rescue the susceptibility and slow stomatal closure of the *cngc16‐1* mutant in response to salt.

### *OsCNGC16* promotes ROS homeostasis in response to salt stress

**Figure 5:**
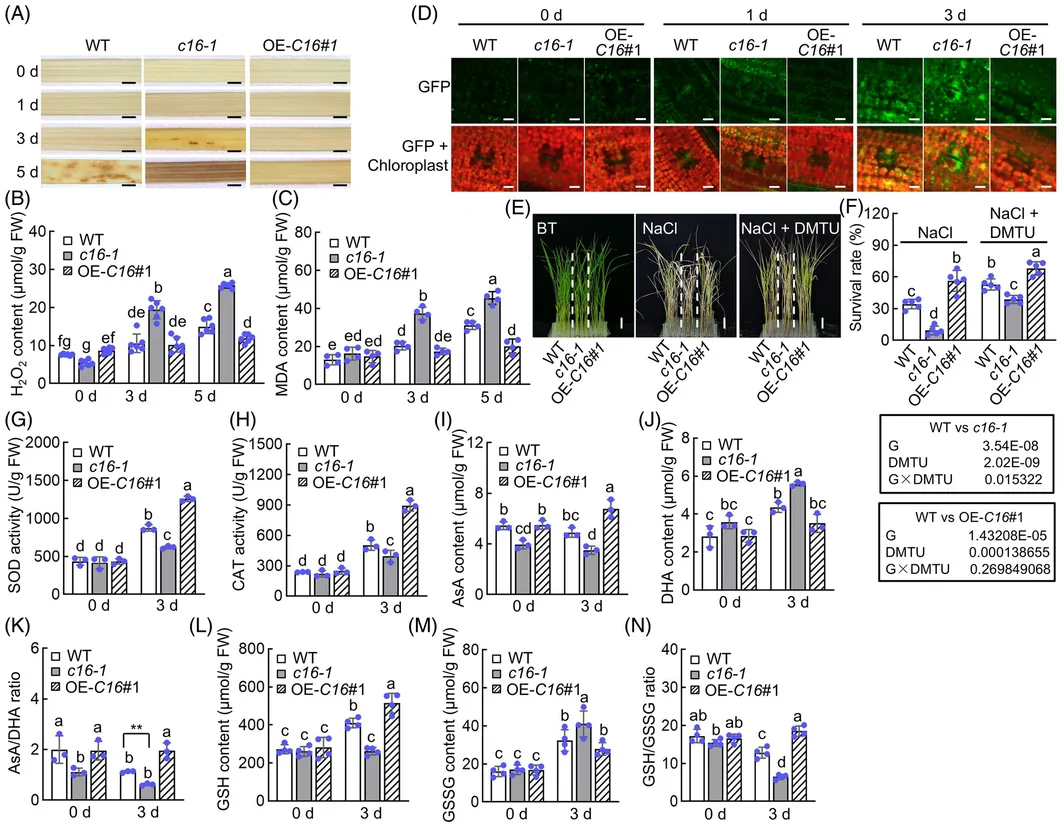
*OsCNGC16* promotes reactive oxygen species (ROS) scavenging under salt treatment. (A–D) DAB staining (A), H_2_O_2_ content (B), MDA content (C), and H2DCFDA staining (D) in the WT, *c16‐1*, and OE‐*C16*#1 plants before and after treatment of 130 mM NaCl. Scale bars, 2 cm in (A) or 10 μm in (D). (E, F) Morphological phenotypes (E) and survival rates (F) of the WT, *c16‐1* and OE‐*C16*#1 plants after treatment of 130 mM NaCl for 8 days with or without 1 mM DMTU. Photographs were taken BT and after recovery. Scale bars, 2 cm. Insert shows multiway‐ANOVA analysis of genotype (G; WT versus *c16‐1* or OE‐*C16*#1) and DMTU treatment (NaCl versus NaCl + DMTU). (G–N) SOD activity (G), CAT activity (H), AsA content (I), DHA content (J), AsA/DHA ratio (K), GSH content (L) GSSH content (M) and GSH/GSSH ratio (N) in the WT, *c16‐1*, and OE‐*C16*#1 plants before and after treatment of 130 mM NaCl. Shown are the means ± SD from at least three biological replicates. Letters in (B, C, F–N) indicate significant differences at *P* < 0.01 by Duncan's multiple range test via ANOVA.

ROS are important signaling molecules under stresses and salt stress can induce accumulation of ROS and malondialdehyde (MDA) that cause damage to plant tissues (Sun et al., 2025; Wang et al., 2025). We detected hydrogen peroxide (H_2_O_2_) and MDA accumulation in the leaf tissues of *cngc16‐1*, OE‐*OsCNGC16*#1, and WT after exposure to 130 mM NaCl. No significant differences in H_2_O_2_ accumulation and MDA content were observed among these genotypes before NaCl treatment (Figure 5A,B). H_2_O_2_ and MDA contents increased in all genotypes after salt treatment, and was significantly higher in *cngc16‐1* but lower in OE‐*OsCNGC16*#1 compared to WT after 130 mM NaCl treatment (Figure 5A–C). Since H_2_O_2_ can induce stomatal closure (Neill et al., 2002) and the three *OsCNGC* genes positively regulate salt‐induced stomatal closure, we examined H_2_O_2_ accumulation in guard cells using H2DCFDA fluorescence dye. The *cngc16‐1* mutant accumulated more H_2_O_2_, while the OE‐*OsCNGC16*#1 plants accumulated less H_2_O_2_ in the guard cells, than those of the WT plants after 130 mM NaCl treatment (Figure 5D). We further analyzed effect of ROS accumulation on salt tolerance of the *cngc16‐1* mutant and OE‐*OsCNGC16*#1 plants. Rice seedlings were treated with 130 mM NaCl solution with or without ROS scavenger dimethylthiourea (DMTU). An increased salt tolerance was observed in all genotypes after DMTU treatment (Figure 5E,F). Further G × E analysis on survival rate showed that significant interaction was found for the *cngc16‐1* genotype and DMTU treatment (Figure 5F), indicating that DMTU treatment partially rescued the salt susceptibility of the *cngc16‐1* mutant. To further assess the role of *OsCNGC16* in ROS scavenging, the enzyme activities of SOD and CAT were detected. After 130 mM NaCl treatment, the activities of SOD and CAT were much higher than non‐treatment control (Figure 5G,H). And these enzyme activities were significantly lower in *cngc16‐1* but higher in OE‐*OsCNGC16*#1 compared to WT after 3 days of 130 mM NaCl (Figure 5G,H). Expression of ROS‐related genes (Alfatih et al., 2020; Chen, Zhang, et al., 2024; Vighi et al., 2016; Wang, Zhao, et al., 2023) was analyzed in *OsCNGC16* transgenic and WT plants before and after salt treatment. Under normal conditions, *OsRBOHA* had a decreased expression in the *cngc16‐1* mutant, while *OsSOD1* had an increased expression in the OE‐*OsCNGC16*#1 plants compared to WT (Figure S7a,c). After 3 h of salt treatment, *OsRBOHA* and *OsRBOHH* had an increased expression in the *cngc16‐1* compared to WT. *OsSOD1* had a decreased expression in the *cngc16* mutant, while *OsCatA* and *OsCatB* had an increased expression in the OE‐*OsCNGC16*#1 plants compared to WT (Figure S7a–e).

For the AsA‐dehydroascorbate (DHA) cycle plays a crucial role in ROS scavenging under salt stress (Zhang, Gao, et al., 2025), we further detected AsA, DHA, GSH, and oxidized glutathione (GSSG) contents in shoots of WT, *cngc16‐1*, and OE‐*OsCNGC16*#1 plants. Under normal conditions, the ascorbate redox state (AsA/DHA) ratio was significantly lower in *cngc16‐1* than in WT (Figure 5I–K). After 3 days of 130 mM NaCl treatment, the AsA/DHA and GSH/GSSG ratios were significantly lower in *cngc16‐1* but higher in OE‐*OsCNGC16*#1 than in WT (Figure 5I–N). We further analyzed the expression of ROS redox homeostasis genes (Bonifacio et al., 2016; Gao et al., 2026; Hong et al., 2009; Hong & Kao, 2008; Li et al., 2023) in *OsCNGC16* transgenic and WT plants before and after salt treatment. Under normal conditions, *OsGPX5*, *OsAPX3*, *OsMDHAR3*, *OsGR1*, and *OsGR2* had increased expression in the OE‐*OsCNGC16*#1 plants compared to WT (Figure S7g,i,j,l,m). After 3 h of 130 mM NaCl treatment, most of these genes (*OsGPX1*, *OsAPX1*, *OsAPX3*, *OsMDHAR3*, *OsGR1*, and *OsGR2*) had decreased expression in the *cngc16‐1* mutant, while *OsGPX5*, *OsAPX1*, *OsDHAR1*, *OsGR1*, and *OsGR2* had increased expression in the OE‐*OsCNGC16*#1 plants compared to WT (Figure S7f–m). Taken together, *OsCNGC16* plays a critical role in maintaining ROS homeostasis in response to salt stress.

### OsCNGC16 interacts with and be phosphorylated by OsCPK5/13

**Figure 6:**
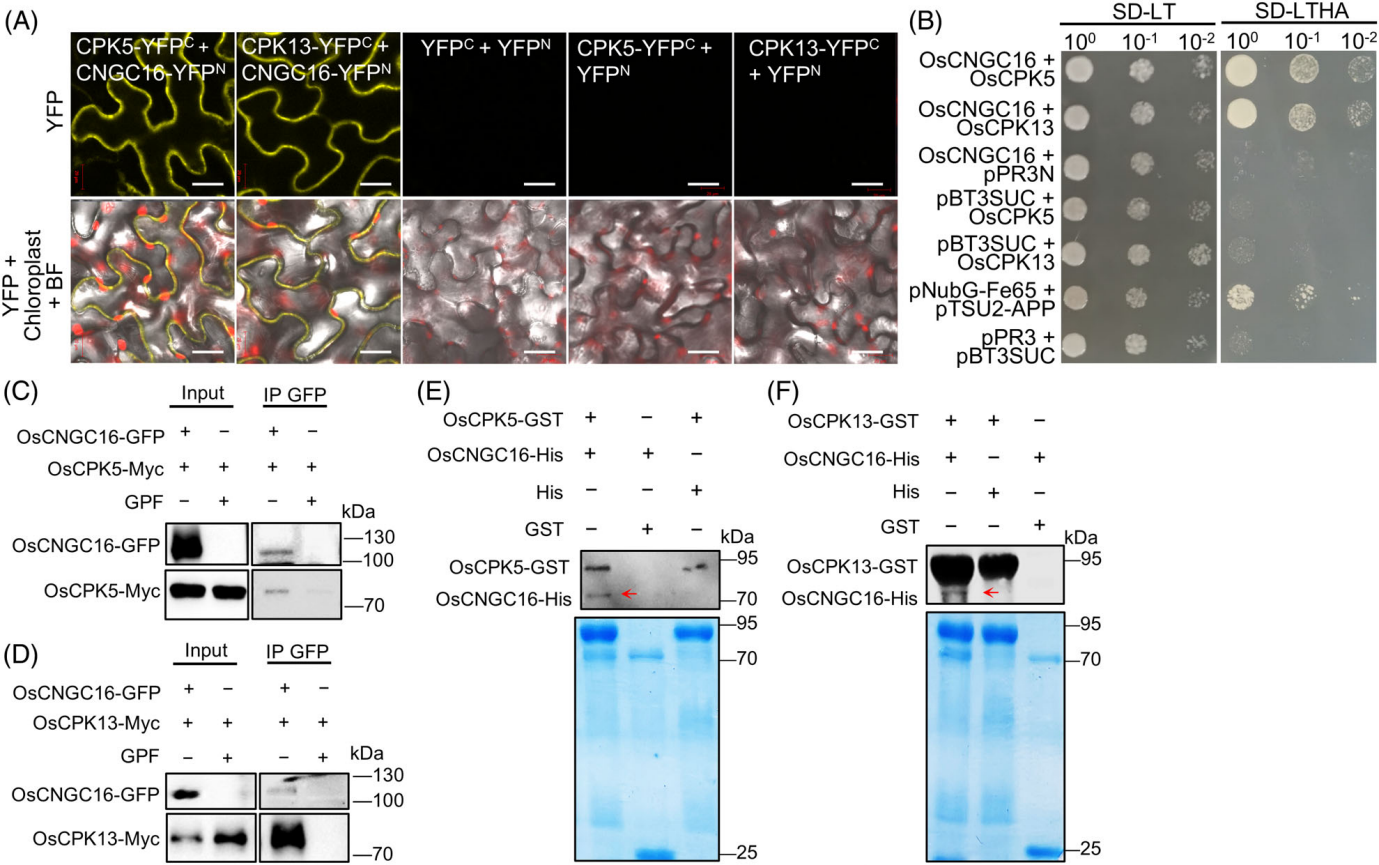
OsCPK5/13 interact with and phosphorylate OsCNGC16. (A) Fluorescent signals from bimolecular fluorescence complementation (BiFC) assays in epidermal cells of *N. benthamiana*. The OsCNGC16 and OsCPK5/13 proteins were fused to the N‐terminal and C‐terminal portions of YFP (YFP^N^ and YFP^C^, respectively) and expressed in *N*. *benthamiana* via agroinfiltration. Scale bars, 20 μm. (B) Yeast grown in dual membrane system yeast two‐hybrid (Y2H) assays. Yeasts adding with 20 mM 3‐AT solution were grown on SD‐Leu‐Trp (SD‐TL) or SD‐Leu‐Trp‐His‐Ade (SD‐TLHA) medium. Yeasts co‐transformed with pNubG‐Fe65 and pTSU2‐APP, and pPR3‐N and pTSU2‐APP were used as a positive and negative control, respectively. (C, D) Co‐immunoprecipitation (Co‐IP) assay showing the interaction between OsCNGC16 and OsCPK5 (C) and OsCNGC16 and OsCPK13 (D) in *N. benthamiana* via agroinfiltration. The immunoprecipitated products were detected with anti‐Myc and anti‐GFP antibodies, respectively. Shown are western blots of total proteins (Input) and IPed proteins. (E, F) Phosphorylation assays of OsCPK5 (E) and OsCPK13 (F) on OsCNGC16. Shown are autoradiograph and coomassie brilliant blue (CBB) staining of gels with proteins from the kinase reaction with (left panel) or without (right panel) Phos‐tag™ BTL‐104 antibody separated on 10% SDS‐PAGE.

To elucidate the regulatory mechanism of the three *OsCNGC* genes in salt tolerance, we sought to identify their interacting proteins. Due to their critical function in regulating ROS homeostasis and Ca^2+^ influx under salt, we focused on OsCPK5/13, which positively regulate salt tolerance and production of ROS during the early response to salt stress in rice (Su et al., 2024). We firstly analyzed the interaction between OsCNGC16 and OsCPK5/13 by BiFC assays. The OsCNGC16 and OsCPK5/13 proteins were each fused to the N‐ and C‐terminal portions of yellow fluorescent protein (YFP^N^ and YFP^C^), respectively, and were co‐expressed in *Nicotiana benthamiana* (*N. benthamiana*) leaves. YFP fluorescence was detected in leaf epidermal cells co‐expressing OsCNGC16‐YFP^N^ and each of OsCPK5‐YFP^C^ and OsCPK13‐YFP^C^ (Figure 6A). In contrast, co‐expressing OsCNGC16‐YFP^N^ and YFP^C^ or OsCPK5/13‐YFP^C^ and YFP^N^ did not yield fluorescent signals (Figure 6A). The results suggest that OsCNGC16 interacts with OsCPK5/13.

We further verified the interaction between OsCNGC16 and OsCPK5/13 using dual membrane Y2H assays (Thaminy et al., 2004). The coding sequences (CDSs) of *OsCPK5/13* and *OsCNGC16* were cloned into the prey vector pPR3‐N and the bait vector pBT3‐SUC, respectively, and each pair of prey and bait constructs were co‐transformed into yeast strain NMY51. Although all yeast cells harboring two constructs grew on SD‐Leu‐Trp medium, only yeast cells harboring the pairs of different proteins, including OsCNGC16‐pBT3‐SUC and OsCPK5‐pPR3‐N and OsCNGC16‐pBT3‐SUC and OsCPK13‐pPR3‐N, grew on selective SD‐Leu‐Trp‐His‐Ade medium (Figure 6B).

The interactions between OsCNGC16 and OsCPK5/13 were further supported by Co‐IP assays. Pairs of proteins with different tags were transiently expressed in *N. benthamiana*, including OsCNGC16‐GFP with OsCPK5‐Myc and OsCNGC16‐GFP with OsCPK13‐Myc. OsCNGC16‐GFP was each detected in fractions immunoprecipitated by anti‐Myc antibody when co‐expressed with OsCPK5‐Myc and OsCPK13‐Myc, respectively, but not with Myc tag alone (Figure 6C,D). Together, these findings confirm that OsCNGC16 interacts with OsCPK5/13 in yeasts and in plant cells.

We further tested whether OsCNGC16 could be directly phosphorylated by OsCPK5 and OsCPK13. OsCPK5 and OsCPK13 proteins were recombinantly expressed and purified as GST‐tagged proteins and were then incubated with purified OsCNGC16‐His protein in the protein kinase assay. Phosphorylation by OsCPK5/13‐GST was detected on itself as well as on OsCNGC16‐His (Figure 6E,F). These data indicate that OsCNGC16 could be direct phosphorylation targets of OsCPK5/13.

## DISCUSSION

**Figure 7:**
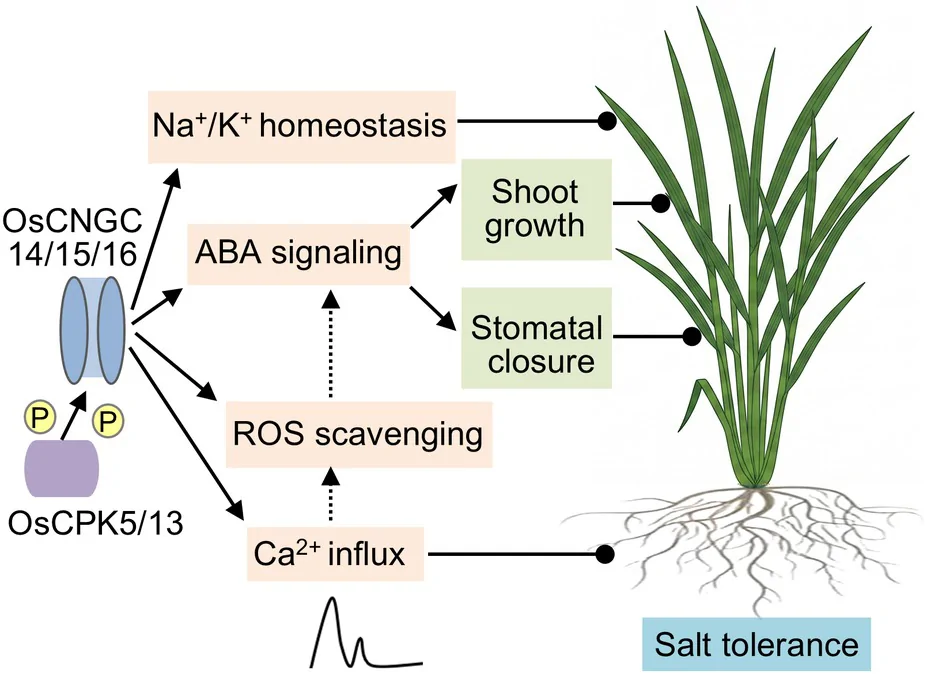
Working model depicting the roles of OsCNGC14/15/16 in regulating rice salt tolerance. OsCNGC14/15/16 proteins phosphorylated by OsCPK5/13 to promote Ca^2+^ influx in root to positively regulate salt tolerance, which also play essential roles in Na^+^/K^+^ homeostasis and reactive oxygen species (ROS) scavenging in leaves to enhance salt tolerance. Meanwhile, OsCNGC14/15/16 promote stomatal closure through ABA signaling pathway for surviving under salt stress. Additionally, OsCNGC14/15/16 regulate shoot growth under ABA treatment, which may also contribute to salt tolerance. Dark lines indicate regulation shown from this study. Dotted lines indicate hypothesized process regulation.

Our previous studies have characterized functions of *OsCNGC14/15/16* genes in stomatal closure and Ca^2+^ influx in response to heat, chilling, drought and the stress hormone ABA, and also in tolerance to heat, chilling and drought (Cui et al., 2020; Luo et al., 2025). However, the role of *OsCNGC14/15/16* genes in salt stress response has not been elucidated. This study identifies *OsCNGC14/15/16* genes also as positive regulators of salt tolerance by controlling stomatal closure, Na^+^/K^+^ homeostasis, Ca^2+^ influx, ROS scavenging and ABA signaling (Figure 7). Mutations of these *OsCNGC* genes lead reduced tolerance, slower stomatal closure, decreased Ca^2+^ influx, higher Na^+^/K^+^ ratio, and higher ROS accumulation in response to salt (Figures 1, 2, 3 and 5). Overexpression of *OsCNGC16* confers increased Ca^2+^ influx, decreased Na^+^/K^+^ ratio, lower ROS accumulation, and enhanced tolerance to salt stress (Figures 2, 3 and 5; Figure S3). In addition, exogenous ABA or DMTU can fully or partially rescue the salt susceptibility of the *cngc16‐1* mutant (Figures 4 and 5). Furthermore, OsCNGC16 might be potential target of OsCPK5/13 in regulating salt tolerance as it can interact with and be phosphorylated by OsCPK5/13 (Figure 6). In addition to previously reported regulating stomatal closure and Ca^2+^ influx, this study characterized new mechanisms of *OsCNGC14/15/16* in salt tolerance, including maintaining Na^+^/K^+^ homeostasis and promoting ROS scavenging. This study also discovered a novel CNGC‐CPK interaction module in response to multiple abiotic stresses in rice (Figure 7).

Under salt stress, ABA‐mediated stomatal closure is an important mechanism and ROS including H_2_O_2_ are proposed to function as second messengers in ABA signaling in guard cells (Bright et al., 2006; Hua et al., 2012; Köhler et al., 2003; Zhao, Zhang, Liu, et al., 2021). Ca^2+^ signals potentially activate Ca^2+^ sensors to promote ABA and ROS accumulation, while ABA can also activate some kinase activity leading to H_2_O_2_ generation (Shi et al., 2012; Sirichandra et al., 2009; Wang, Shen, et al., 2023). Furthermore, ABA‐induced ROS accumulation activates Ca^2+^ channels and promotes stomatal closure (Pei et al., 2000). So, ABA, Ca^2+^, and ROS signaling constitute a feedback regulatory network that coordinates salt stress adaptation in plants. Salt‐induced Ca^2+^ influx is largely dependent on ABA signaling, as it is strongly reduced in the *snrk2s* mutants (Lin et al., 2025). ABA‐activated SnRKs phosphorylate RBOHF to promote production of H_2_O_2_ under salt stress (Sirichandra et al., 2009). ABA and Ca^2+^ signals can maintain ROS homeostasis by activating RBOHF through SnRK2.6 and calcineurin B‐like protein‐interacting protein kinase 11/26 (CIPK11/26) signaling modules, whereas ABA insensitive 1 (ABI1) plays a repressive role in ROS homeostasis in response to salt (Han et al., 2019). This study shows that the same *CNGC* genes positively regulate salt tolerance by co‐regulating stomatal closure, Ca^2+^ influx, ROS and ABA signaling in plants. Mutants of *OsCNGC14/15/16* genes had decreased Ca^2+^ influx and higher ROS accumulation (Figures 3 and 5), and also had slower stomatal closure under salt and ABA treatment (Figure 2A; Luo et al., 2025). Defects in stomatal closure and salt tolerance in the *OsCNGC16* mutant could be rescued by exogenous ABA treatment (Figure 4). These results suggest that *OsCNGC14/15/16* may contribute to ABA‐induced stomatal closure in regulating salt tolerance (Figure 7), which will need to be further investigated such as determining endogenous ABA accumulation in response to salt stress. Additionally, *OsCNGC14/15/16* can promote shoot growth under ABA treatment, which may also contribute to salt tolerance (Figure 7).

Under salt stress, the concentration of Ca^2+^ in plant roots increases, leading to the production of ROS, which inhibit the transpiration of stomata in the leaves through ABA pathway (Ma, Chen, et al., 2025; Medeiros et al., 2020; Yang et al., 2022). *RBOH* genes are regulated by Ca^2+^ signals, which are tightly associated with early ROS generation (Kurusu et al., 2015). We showed that *OsRBOHA* and *OsRBOHH* had an increased expression in *cngc16‐1*, and higher ROS accumulation was observed in *cngc16‐1* compared to WT after salt treatment (Figure 5A,B,D; Figure S7a,b). The *cngc16‐1* mutant had lower SOD and CAT activities and AsA/DHA and GSH/GSSG ratios compared to WT after salt treatment (Figure 5G–N). Exogenous ROS scavenger DMTU treatment could partially rescue the salt susceptibility of the *cngc16‐1* mutant (Figure 5E,F). These results suggest that *OsCNGC16* may act as a negative regulator of RBOH‐dependent early ROS production. Meanwhile, *OsCNGC16* promotes stomatal closure through the ABA pathway and may also positively regulate Ca^2+^‐mediated ROS scavenging (Figure 7), including increasing antioxidant enzyme activity and redox‐related metabolite content. Future studies will test these hypotheses to identify the relationship of Ca^2+^ and ROS signals regulated by *OsCNGC16* under salt stress.

Besides Ca^2+^ channels, Ca^2+^‐binding proteins are also reported to be involved in salt tolerance (Jin et al., 2025). In Arabidopsis, AtCDPK16 promotes salt‐responsive root growth through phosphorylating glutamate receptor‐like protein 3.6 (AtGLR3.6; Silamparasan et al., 2023). *AtCPK3*, *AtCPK6*, *AtCPK12*, and *AtCPK27* genes positively regulate salt tolerance (Mehlmer et al., 2010; Xu et al., 2010; Zhang et al., 2018; Zhao et al., 2015). AtCaM4 interacts with AtSOS2 and activates the SOS pathway to positively regulate salt tolerance (Zhang, Li, et al., 2025). In rice, *OsCPK21* positively regulates ABA signaling and salt tolerance through phosphorylating Os14‐3‐3 (OsGF14e; Chen, Zhou, et al., 2017). OsCPK5 and OsCPK13 proteins positively regulate salt tolerance by directly activating OsMPK3/6 kinases (Su et al., 2024). However, the relationship between these Ca^2+^‐binding proteins and CNGCs needs to be further explored in response to salt stress. This study shows that OsCNGC16 interacts with and is phosphorylated by OsCPK5/13 (Figure 6). Future study should determine the functional significance of the interaction between OsCNGC16 and OsCPK5/13, for example identifying and generating OsCNGC16 mutations of the specific phosphorylation sites in *cngc16‐1* background to test their ability to rescue salt susceptibility of the *cngc16‐1* mutant.

Salt stress influences Na^+^/K^+^ balance to maintain ionic homeostasis in plants (Raddatz et al., 2020; Van Zelm et al., 2020; Zhao, Zhang, Liu, et al., 2021). *CNGCs* genes have also been characterized to transport K^+^ and Na^+^ under salt stress. In *Hordeum vulgare*, *HcCNGC2‐3* is activated by the coincident presence of Na^+^ and K^+^ and exhibits non‐selective permeability to both ions (Mori et al., 2018). In Arabidopsis, *AtCNGC10* negatively regulates salt tolerance and is involved in Na^+^/K^+^ transport in roots (Guo et al., 2010). We showed that Na^+^ content and Na^+^/K^+^ ratio were significantly higher in *cngc16‐1* but lower in OE‐*OsCNGC16*#1 compared to WT (Figure 3A–C), and lots of Na^+^/K^+^ transporter genes a decreased expression in *cngc16‐1* and increased expression in OE‐*OsCNGC16*#1 compared to WT (Figure 3D–I). These results indicate that *OsCNGC16* may positively regulate Na^+^/K^+^ transporter gene expression to export Na^+^ and confer salt tolerance (Figure 7). However, the mechanisms of *OsCNGC14/15/16* in maintaining Na^+^/K^+^ homeostasis in response to salt stress need further characterization.

In conclusion, our findings reveal that three closely related *OsCNGC14/15/16* genes play a crucial role in salt tolerance through coordinating stomatal closure, Ca^2+^ influx, ABA signaling, Na^+^/K^+^, and ROS homeostasis in rice. Our study shows that the same *CNGC* genes can have a general function in multiple pathways rather than a unique function in salt stress response. Our study will enhance mechanistic understanding of salt stress responses in plants, and the multifaceted roles of *OsCNGC14/15/16* highlight the importance of *CNGC* genes in salt stress responses.

## MATERIALS AND METHODS

### Plasmid construction and plant materials

For bimolecular fluorescent complementation (BiFC) analysis, the CDSs of *OsCPK5/13* were cloned into the P2YC vector and the CDS of *OsCNGC16* was cloned into the P2YN vector. For co‐immunoprecipitation (Co‐IP) assays, the CDSs of *OsCPK5/13* were cloned into the pCAMBIA1300‐Myc vector and the CDS of *OsCNGC16* was cloned into the phpt‐pSATN1‐GW‐GFP vector. For dual membrane yeast two‐hybrid (Y2H) assays, the CDSs of *OsCPK5/13* and *OsCNGC16* were cloned into the pPR3‐N and pBT3‐SUC vectors, respectively. All the primers used for construct generation are listed in Table S1.

All the mutants of *OsCNGC14/15/16* used in this study were generated and the mutation types were described in our previous studies, including the loss or reduction of function mutants *cngc14‐1*, *cngc14‐2*, *cngc14‐3*, *cngc15‐1*, *cngc15‐2*, *cngc15‐3*, *cngc16‐1*, and *cngc16‐2* (Cui et al., 2020; Luo et al., 2025). The NES‐YC3.6 reporter was introduced into *cngc14‐2*, *cngc15‐1*, and *cngc16‐1* by crossing, respectively (Cui et al., 2020; Luo et al., 2025). The *cngc14 cngc16* and *cngc15 cngc16* double mutants were obtained by crossing *cngc14‐1* with *cngc16‐1*, and *cngc15‐1* with *cngc16‐1*, respectively (Luo et al., 2025). The *OsCNGC14/15/16* complementation, *OsCNGC16* overexpression and *pOsCNGC16::GUS* transgenic lines were generated and identified previously (Luo et al., 2025).

### Plant growth and treatment conditions

Seeds were disinfected with 5% bleach for 10 min, washed three times with water, and then soaked in water for 3–5 days at 26°C in growth chambers for germination. For hydroponic growth, germinated seeds were transferred to a 96‐well plate with bottom cut and were cultured in water till the emergence of the third leaf and then in Yoshida nutrient solution (Yoshida et al., 1976).

Salt tolerance assays were performed as described previously (Yuenyong et al., 2018) with minor modifications. Briefly, hydroponically grown seedlings of three‐leaf stage were treated at 130 mM NaCl for 7–8 days followed by a 7‐days recovery growth in normal condition. Seedlings after recovery with newly formed leaves were defined to be surviving. Each set of experiments had three replicate plates (at least 24 individual seedlings), and three biological replicates were performed for each analysis. For assays of shoot and root length, hydroponically grown seedlings of 2‐days‐old were treated at 120 mM NaCl or ABA (1 or 2 μM) for 7 days (Li et al., 2022). The shoot and root length from 18 individual plants for each genotype was measured. Three biological replicates were performed for each analysis. Salt tolerance assays pretreated with ABA or DMTU were performed as described previously (Chen et al., 2022; Zhang et al., 2021) with minor modifications. Briefly, hydroponically grown seedlings of three‐leaf stage were sprayed with 100 μM ABA or 1 mM DMTU until the leaves were wet, and then treated at 130 mM NaCl or 130 mM NaCl containing 1 mM DMTU for 7 or 8 days followed by a 7‐days recovery growth, respectively. Each set of experiments had three replicate plates (at least 24 individual seedlings), and three biological replicates were performed for each analysis.

### Histochemical staining assays

GUS staining was performed as previously described (Jefferson et al., 1987). In brief, 10‐days‐old seedlings were collected before or after 24 h of 130 mM NaCl treatment and then incubated in GUS staining solution (50 mM NaPO4 buffer [pH 7.0], 0.1% Triton X‐100, 1 mM X‐Gluc). H_2_O_2_ accumulation was visualized by 3, 3′‐diaminobenzidine (DAB) and 2,7‐dichlorodihydrofluorescein diacetate (H2DCFDA) staining as previously reported (Charng et al., 2007; Huang et al., 2009). The third leaves of three‐leaf‐stage hydroponically grown seedlings were used before or after 1‐, 3‐, or 5‐days treatment of 130 mM NaCl. Images of DAB or H2DCFDA staining were collected from a dissecting or a ZEISS LSM 780 confocal microscope (ZEISS Microsystems, Wetzlar, Hessen, Germany), respectively.

### Stomatal aperture assays

Stomata were observed on epidermal impressions of the third leaf of seedlings as described previously (Wu & Zhao, 2017). Briefly, nail polish was applied to the leaf surface before leaves were cut off from the plant. Dried nail polish was peeled from leaf and placed on slides with water to hydrate the peel. This epidermal impression was then observed under an Olympus BX53 microscope. Stomatal apertures were calculated as inner width/inner length, and 50 stomata from four individual leaves, each from a different seedling, were analyzed for each genotype/time point.

### Measurements of H_2_O_2_ , MDA, antioxidant enzyme activity, and ratios of AsA/DHA, GSH/GSSG, and Na^+^/K^+^

0.5 g of fresh leaf tissues were ground into fine powder using liquid nitrogen, and then used for physiological and biochemical measurements. Leaves collected from three seedlings were considered as one biological replicate, and three biological replicates were used for each analysis. The contents of MDA, H_2_O_2_, AsA, DHA, GSH, GSSG, Na^+^, and K^+^ contents were measured as described previously (Deng et al., 2015; Li et al., 2025; Qiu et al., 2020). The activities of SOD and CAT were measured as described previously (Chen et al., 2020). Quantitatively measurements were performed using assay kits (Solarbio, Beijing, China) according to the manufacture's instruction.

### RNA isolation and gene expression assays

Total RNAs of leaves were extracted using TsingZol (TSP401, Tsingke, Beijing, China) according to the manufacturer's protocol. 500 ng of total RNAs were reverse transcribed into cDNA using HiScript III RT SuperMix (R323, Vazyme, Nanjing, China). For gene expression analysis, leaves from three‐leaf stage hydroponically grown seedlings of WT, *cngc16‐1*, and *OE‐OsCNGC16* plants were collected before and after different time points of 130 mM NaCl treatment. The qRT‐PCR assays were performed using AceQ® qPCR SYBR® Green Master Mix (Q111, Vazyme, Nanjing, China) and the Bio‐Rad CFX96 Real‐Time PCR system (BioRad Laboratories, Hercules, CA, USA). *OsActin1* (LOC_Os03g61970) was used as the reference gene for expression. Primers for qRT‐PCR are listed in Table S1.

### Transient expression and microscopy analysis

Transient expression in *N. benthamiana* plants was carried out via *Agrobacterium*‐mediated transformation (Nishimura et al., 2006) with minor modifications. Cells of *A*. *tumefaciens* GV3101 carrying desired constructs were cultured, washed, and suspended in infiltration buffer (10 mM MgCl_2_, 10 mM MES, 150 μM acetosyringone) to a final OD_600_ of 0.5. Suspended cells were injected on the abaxial side of leaves on 4‐week‐old *N. benthamiana* plants. Signals from transformed cells, usually fluorescence signals, were observed under a ZEISS LSM 780 confocal microscope (ZEISS Microsystems, Wetzlar, Hessen, Germany). GFP, YFP, and chlorophyll were excited at 488, 514, and 488 nm, respectively. Emission is captured at 509–530 nm for GFP, 525–550 for YFP, and 650–700 nm for chlorophyll. ZEISS Microsystem was used to analyze images.

### DUAL membrane system Y2H assays

Y2H assays were performed through the DUAL membrane system (Dualsystems Biotech, P01201‐P01229). The CDSs were cloned into the prey pBT3‐SUC or bait pPR3‐N vectors and co‐transformed pairwise into yeast strain NMY51 (Xu et al., 2017). Yeast cells were grown on SD medium lacking Leu and Trp (SD‐Leu‐Trp) or lacking Leu, Trp, His, and Ade (SD‐Leu‐Trp‐His‐Ade) at 30°C for 3 days. 3‐AT solution (20 mM) was added to the yeast colonies grown on SD‐Leu‐Trp‐His‐Ade to inhibit self‐activation. Yeasts co‐transformed with pNubG‐Fe65 and pTSU2‐APP were used as a positive control, and co‐transformed with pPR3‐N and pTSU2‐APP were used as a negative control.

### Co‐IP assays

Co‐IP assays were performed by transient expression in *N. benthamiana*. OsCNGC16‐GFP and OsCPK5‐Myc, and OsCNGC16‐GFP and OsCPK13‐Myc were co‐expressed in *N. benthamiana* leaf epidermal cells, respectively. Total proteins were extracted using extraction IP buffer (50 mM Tris–HCL, pH = 7.5, 150 mM NaCl, 0.2% (v/v) NP‐40, 5 mM DTT, and 1× protease inhibitor cocktail) and then incubated with anti‐GFP antibody agarose beads (MBL, New Jersey, USA) for 3 h at 4°C. Beads that contained the GFP immunoprecipitated proteins were washed five times with washing buffer (50 mM Tris–HCl, pH = 7.5, 150 mM NaCl, 0.1% NP‐40) and mixed with SDS loading buffer followed by incubation at 95°C for 10 min. Supernatants were then separated on 10% SDS‐PAGE gels and immunoblotted with anti‐GFP antibody (Sigma‐Aldrich, USA, dilution 1:3000) and anti‐Myc antibody (Cell Signaling Technology, Danvers, MA, USA, dilution 1:3000).

### *In vivo* phosphorylation assays

Recombinant protein expression and purification in *E. coli* was performed as described previously (Briand et al., 2016; Scheich et al., 2003) with minor modifications. Fusion proteins of OsCPK5‐GST and OsCPK13‐GST were induced by 0.5 mM isopropyl‐β‐_D_‐thiogalactoside (IPTG) in BL21 (DE3) at 16°C overnight. OsCNGC16‐His fusion protein was induced by 0.3 mM IPTG in BL21 (Rosetta) at 37°C overnight. OsCPK5/13‐GST or OsCNGC16‐His fusion proteins were purified using a Q‐Sepharose column with GST bind™ resin or Ni Sepharose™ fast flow agarose beads (GE Healthcare), respectively. 1 μg of purified recombinant OsCPK5‐GST or OsCPK13‐GST fusion proteins was incubated with 5–10 μg purified recombinant OsCNGC16‐His substrate in a kinase reaction buffer (10 × PKA and 50 mM ATP) (Solarbio, Beijing, China) at 37°C for 1 h. Proteins from the reaction were separated by SDS‐PAGE, and the phosphorylation signal was detected using the Phos‐tag™ BTL‐104 antibody.

### Ca^2+^ imaging

FRET‐based Ca^2+^ imaging of stomata was performed as described previously (Krebs et al., 2012; Luo et al., 2025). Roots of 7‐day‐old seedlings were cut into 1.5 cm in length and put on the dishes with 25 mL of half strength Murashige and Skoog (1/2 MS) buffer. Incubation buffer (5 mM KCl, 50 mM CaCl_2_, 10 mM MES‐Tris, pH = 6.2) was applied at 50 μL to a glass slide holding a root slice. For salt treatment, the buffer contains additional 130 mM NaCl. YC3.6 fluorescence was analyzed using a confocal laser scanning microscope (Zeiss LSM780) described previously (Luo et al., 2025). YFP and CFP fluorescence intensity was monitored by ZEISS Microsystem every 2 s. Cytosol Ca^2+^ level was expressed as R:R0, from YFP/CFP (cpVenus/ECFP) fluorescence ratio (R) and normalized by the ratio at time point zero (R0) as described previously (Krebs et al., 2012). Maximum change in Ca^2+^ level (∆R_peak_) is the difference between R:R0 at the Ca^2+^ peak time point and R:R0 before treatment.

### Statistical analysis

Statistical analysis was performed using the Excel 2021 (Microsoft, USA) and STST (Nanjing Agricultural University, Nanjing, China; Chen, Du, et al., 2017) software. Student's *t*‐test was used for comparisons between two groups, and Duncan's multiple range test via ANOVA was used for multiple group comparisons.

## ACCESSION NUMBERS

DNA sequence data were obtained from the Rice Annotation Project Database (http://rice.plantbiology.msu.edu/index.shtml) according to the following accession numbers: LOC_Os03g55100 (OsCNGC14), LOC_Os01g57370 (OsCNGC15), LOC_Os05g42250 (OsCNGC16), LOC_Os02g46090 (OsCPK5), and LOC_Os04g49510 (OsCPK13).

## AUTHOR CONTRIBUTIONS

SL and BZ conceived and designed the study. LL, XG, YC, and DW performed experiments. LL, XG, and SL analyzed the data. SL, LL, and XG wrote and revised the manuscript. All authors read and approved the contents of this paper.

## CONFLICT OF INTEREST

The authors have no conflicts of interest to declare.

## Supporting information


**Figure S1.** Expression patterns of *OsCNGC14/15/16* in response to salt treatment.
**Figure S2.** Salt tolerance of the *OsCNGC14/15/16* complementation lines.
**Figure S3.** Salt tolerance of the *OsCNGC16* overexpression lines.
**Figure S4.** Growth of the *cngc14/15/16* single and double mutants under salt stress.
**Figure S5.** Effects of exogenous abscisic acid (ABA) on growth of the *cngc14/15/16* single and double mutants.
**Figure S6.** Growth of the *OsCNGC16* overexpression lines under salt stress and ABA treatment.
**Figure S7.** Expression patterns of reactive oxygen species (ROS) homeostasis‐related genes in the *cngc16* and OE‐*OsCNGC16* plants under salt stress.
**Table S1.** List of primers used in this study.

## Data Availability

Processed data are presented in this article. Raw data will be shared upon request.
