# Supplementary material for: Cyclic nucleotide‐gated channels 14, 15, and 16 coordinate multiple signaling pathways to promote salt tolerance in rice
Source: Plant J. 2026 Aug 19;127(4):e71092. doi: 10.1111/tpj.71092 (PMC13489427; doi:10.1111/tpj.71092)
Supplement: Supplementary file 1 [file TPJ-127-0-s001.pdf]

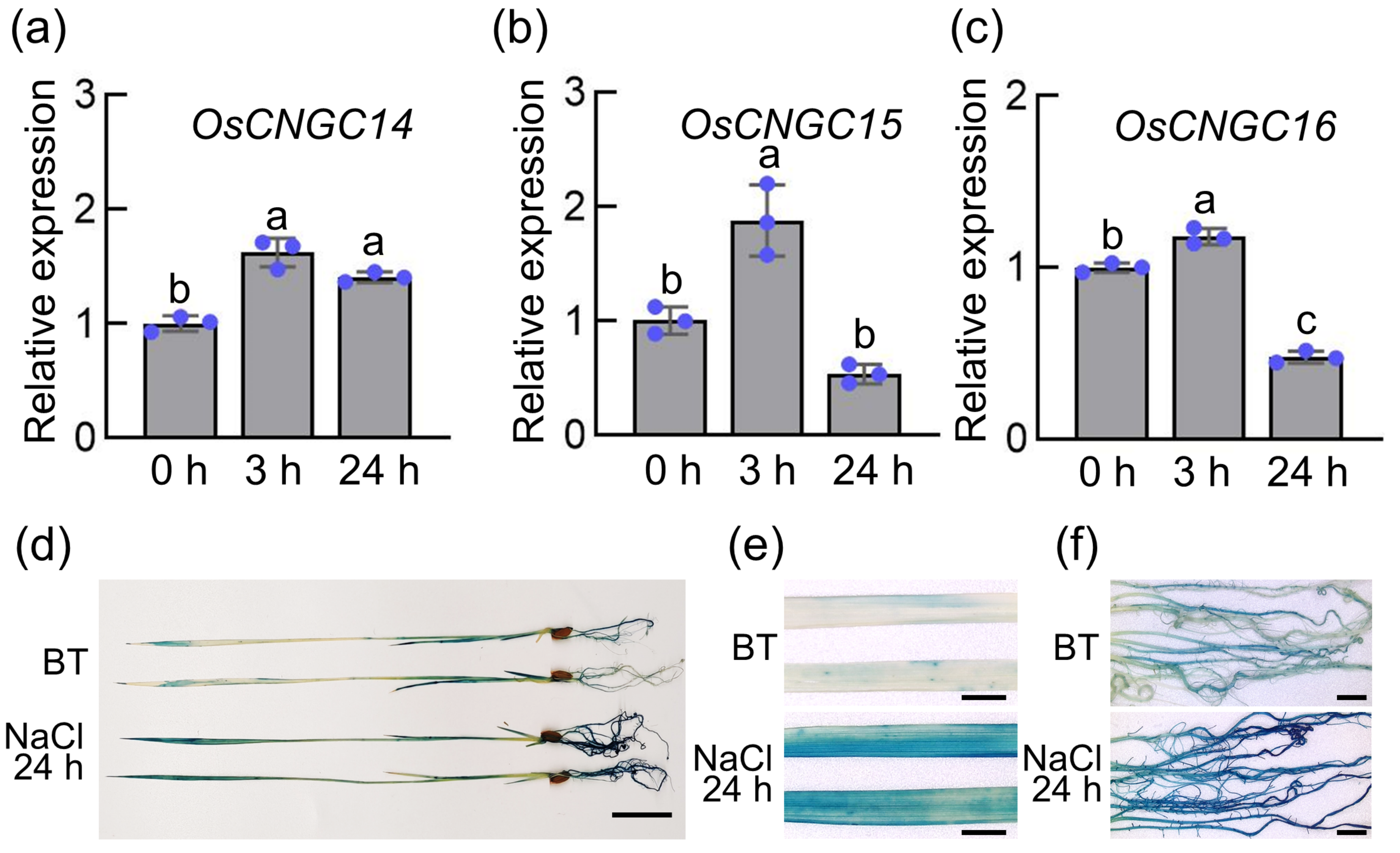

Figure S1. Expression patterns of *OsCNGC14/15/16* in response to salt treatment.

(a-c) Relative expression levels of *OsCNGC14* (a), *OsCNGC15* (b) and *OsCNGC16* (c) in leaf tissues of 2-week-old Nipponbare (NIP) seedlings exposed to 130 mM NaCl for 0 h, 3 h and 24 h. Transcript levels were measured by quantitative real-time PCR (qRT-PCR) and represented as relative to the level of 0 h. *OsActin1* was used as the reference gene. Shown are the means ± standard deviation (SD) from three biological replicates. Letters indicate significant differences at $p < 0.01$ by Duncan's multiple range test via ANOVA. (d-f) GUS staining of the 10-days-old *pOsCNGC16::GUS* transgenic seedlings (d), leaves (e) and roots (f) before treatment (BT) and after 24 h of 130 mM NaCl treatment. Scale bars, 2 cm in (d) and 2 mm in (e, f).

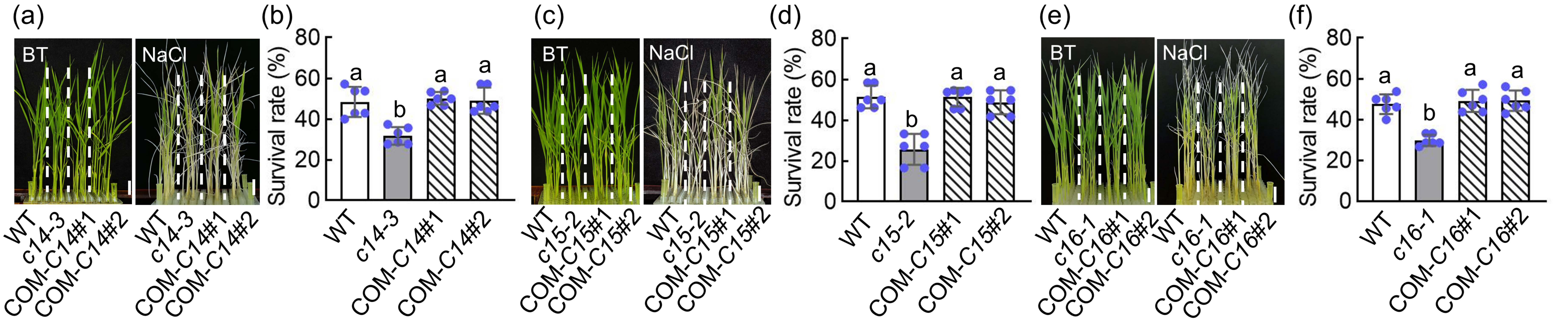

Figure S2. Salt tolerance of the *OsCNGC14/15/16* complementation lines.

Morphological phenotypes (a, c, e) and survival rates (b, d, f) of the complementation (COM) transgenic lines of *OsCNGC14* (COM-*C14*#1 and #2; a, b), *OsCNGC15* (COM-*C15*#1 and #2; c, d) and *OsCNGC16* (COM-*C16*#1 and #2; e, f) seedlings after treatment of 130 mM NaCl for 7 days compared with the wild type (WT) and the *cngc14-3* (*c14-3*), *cngc15-2* (*c15-2*), *cngc16-1* (*c16-1*), respectively. Photographs were taken before treatment (BT) and after recovery. Scale bars, 2 cm. Shown in (b, d, f) are means ± SD from six biological replicates. Letters indicate significant differences at $p < 0.01$ by Duncan's multiple range test via ANOVA.

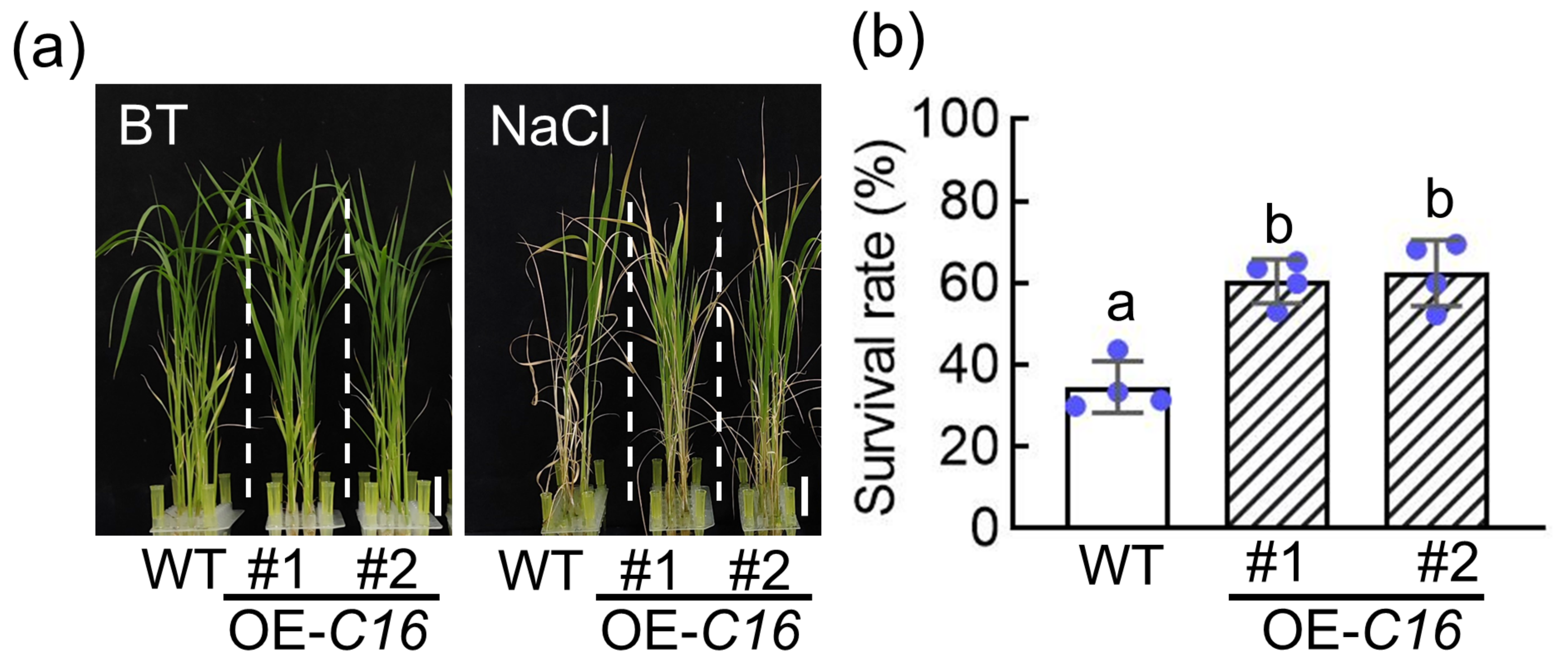

Figure S3. Salt tolerance of the *OsCNGC16* overexpression lines. Morphological phenotypes (a) and survival rates (b) of the WT and two *OsCNGC16* overexpression lines (OE-*C16*#1 and #2) after treatment of 130 mM NaCl for 8 days. Photographs were taken BT and after recovery. Scale bars, 2 cm. Shown are the means ± SD from four biological replicates. Letters indicate significant differences at $p < 0.01$ by Duncan's multiple range test via ANOVA.

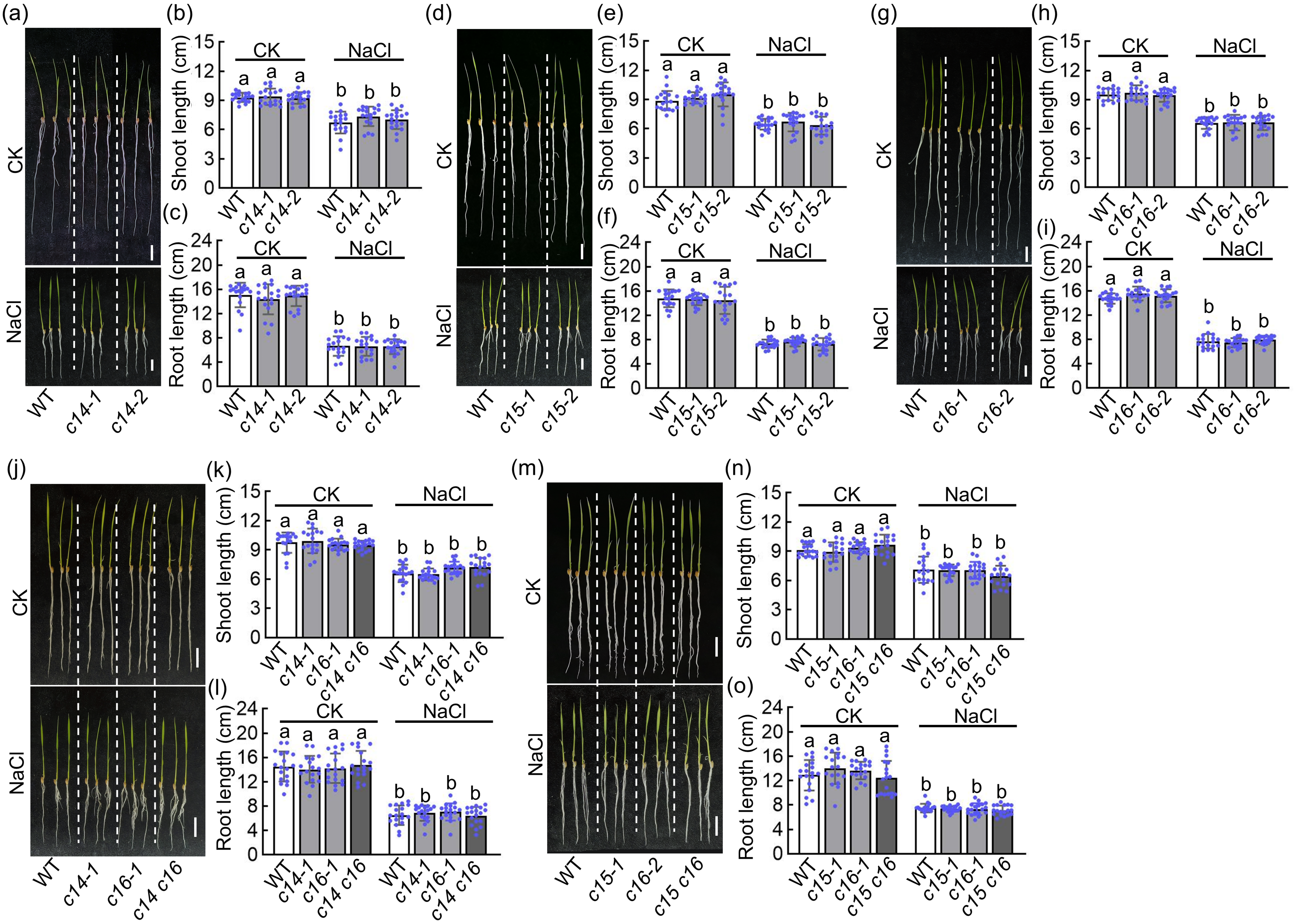

Figure S4. Growth of the *cngc14/15/16* single and double mutants under salt stress.

Morphological phenotypes (a, d, g, j, m), shoot (b, e, h, k, n) and root length (c, f, i, l, o) of the *c14* (a-c), *c15* (d-f), *c16* (g-i), *c14 c16* (j-l) and *c15 c16* (m-o) seedlings after treatments of 120 mM NaCl for 7 days compared with the WT. Scale bars, 2 cm. CK indicates nontreatment control. Shown in (b, c, e, f, h, I, k, l, n, o) are means ± SD from 18 independent plants. Letters indicate significant differences at $p < 0.01$ by Duncan's multiple range test via ANOVA.

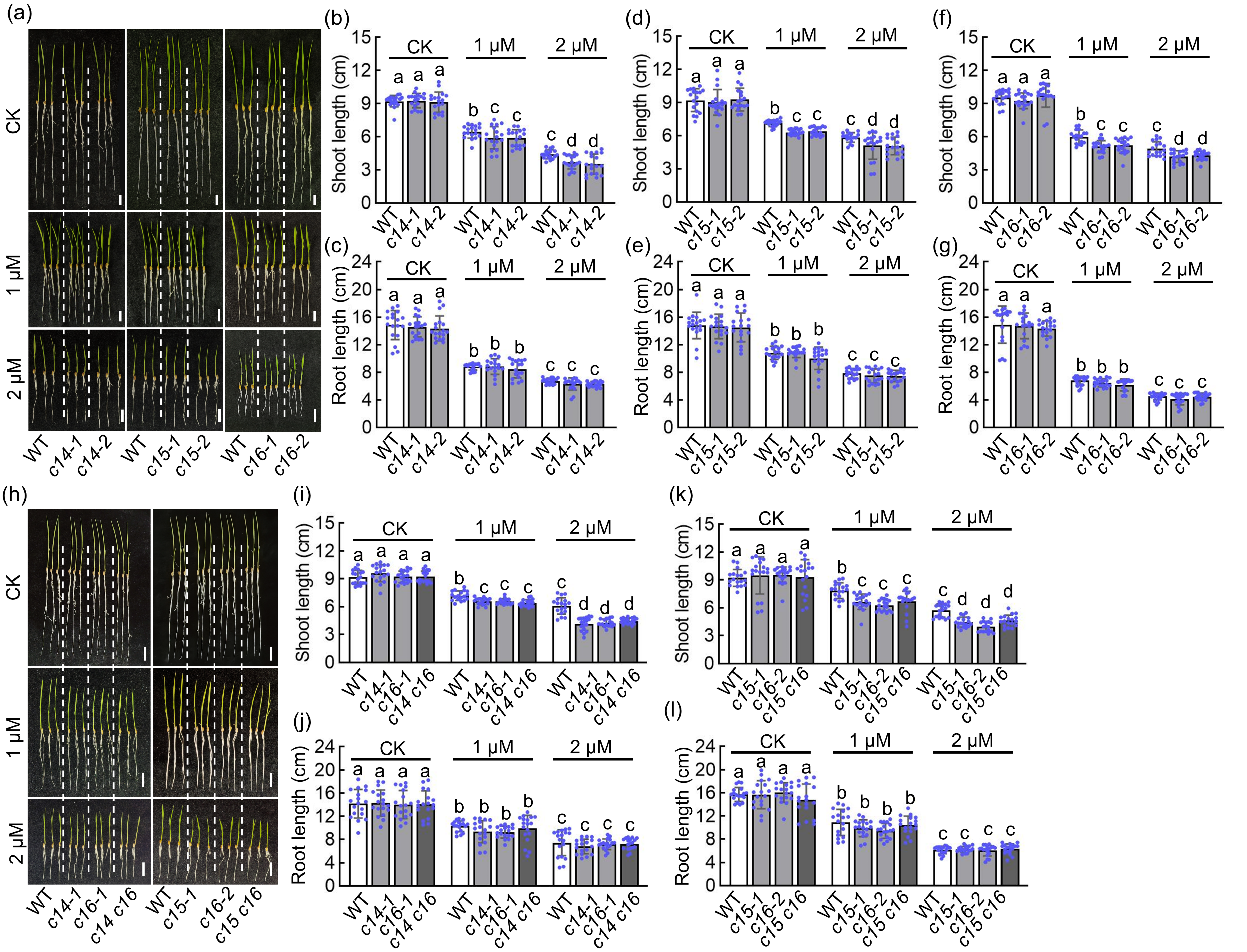

Figure S5. Effects of exogenous ABA on growth of the *cngc14/15/16* single and double mutants.

Morphological phenotypes (a, h), shoot (b, d, f, i, k) and root length (c, e, g, j, l) of the *c14* (b, c), *c15* (d, e), *c16* (f, g), *c14 c16* (i, j) and *c15 c16* (k, l) seedlings after treatments of 1 or 2 μM ABA for 7 days compared with the WT. Scale bars, 2 cm. CK indicates nontreatment control. Shown are means ± SD from 18 independent plants. Letters indicate significant differences at $p < 0.01$ by Duncan's multiple range test via ANOVA.

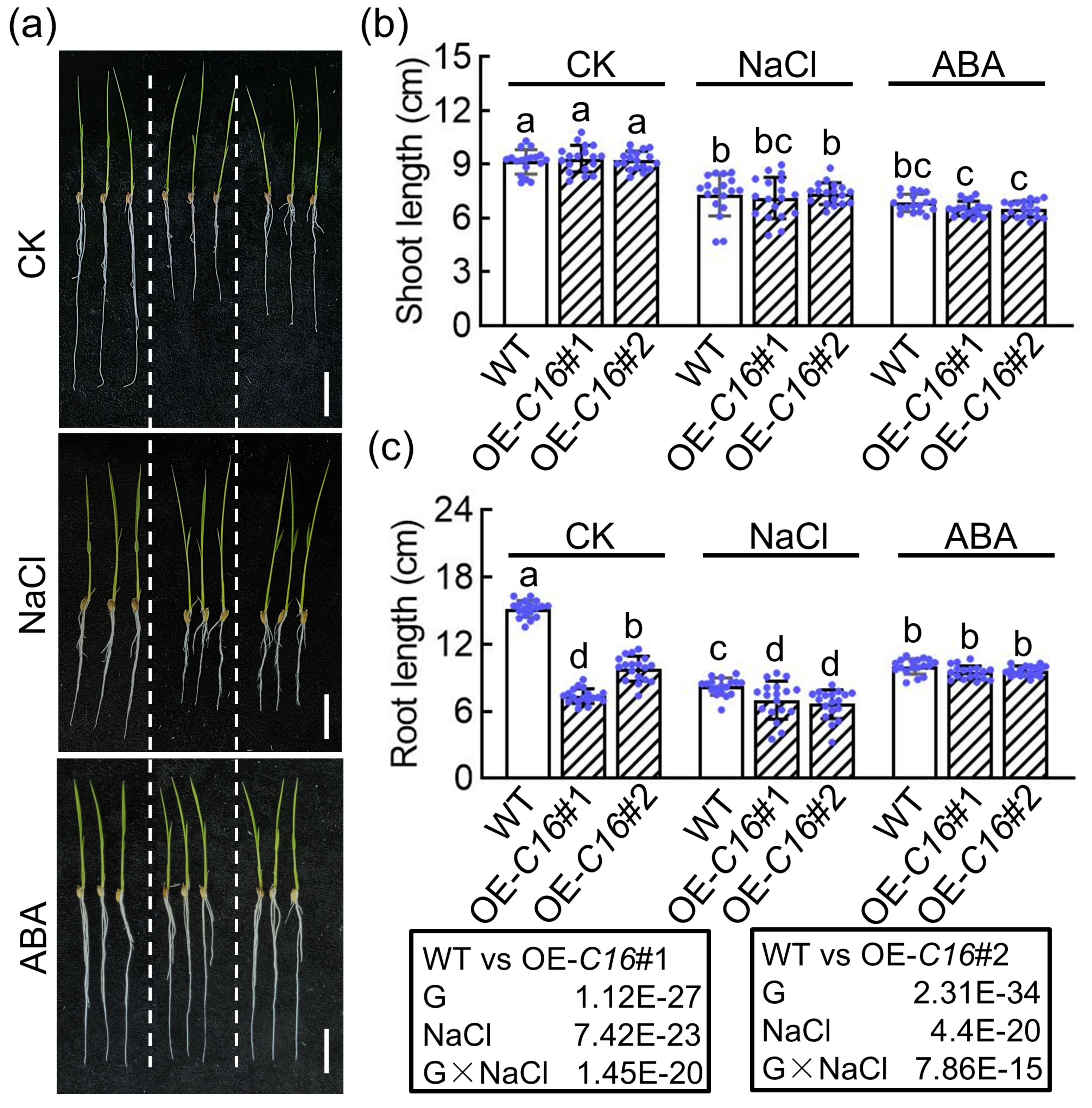

Figure S6. Growth of the *OsCNGC16* overexpression lines under salt and ABA treatments.

Morphological phenotypes (a), shoot (b) and root length (c) of the OE-*OsCNGC16* (OE-*C16*#1 and #2) seedlings after treatments of 120 mM NaCl or 1 μM ABA for 7 days compared with WT. Scale bars, 2 cm. Shown in (b, c) are means ± SD from 18 independent plants. Letters indicate significant differences at $p < 0.01$ by Duncan's multiple range test via ANOVA. Insert shows multiway-ANOVA analysis of genotype (G; WT versus the OE-*C16*#1 or #2) and salt treatment (CK, nontreatment control versus NaCl).

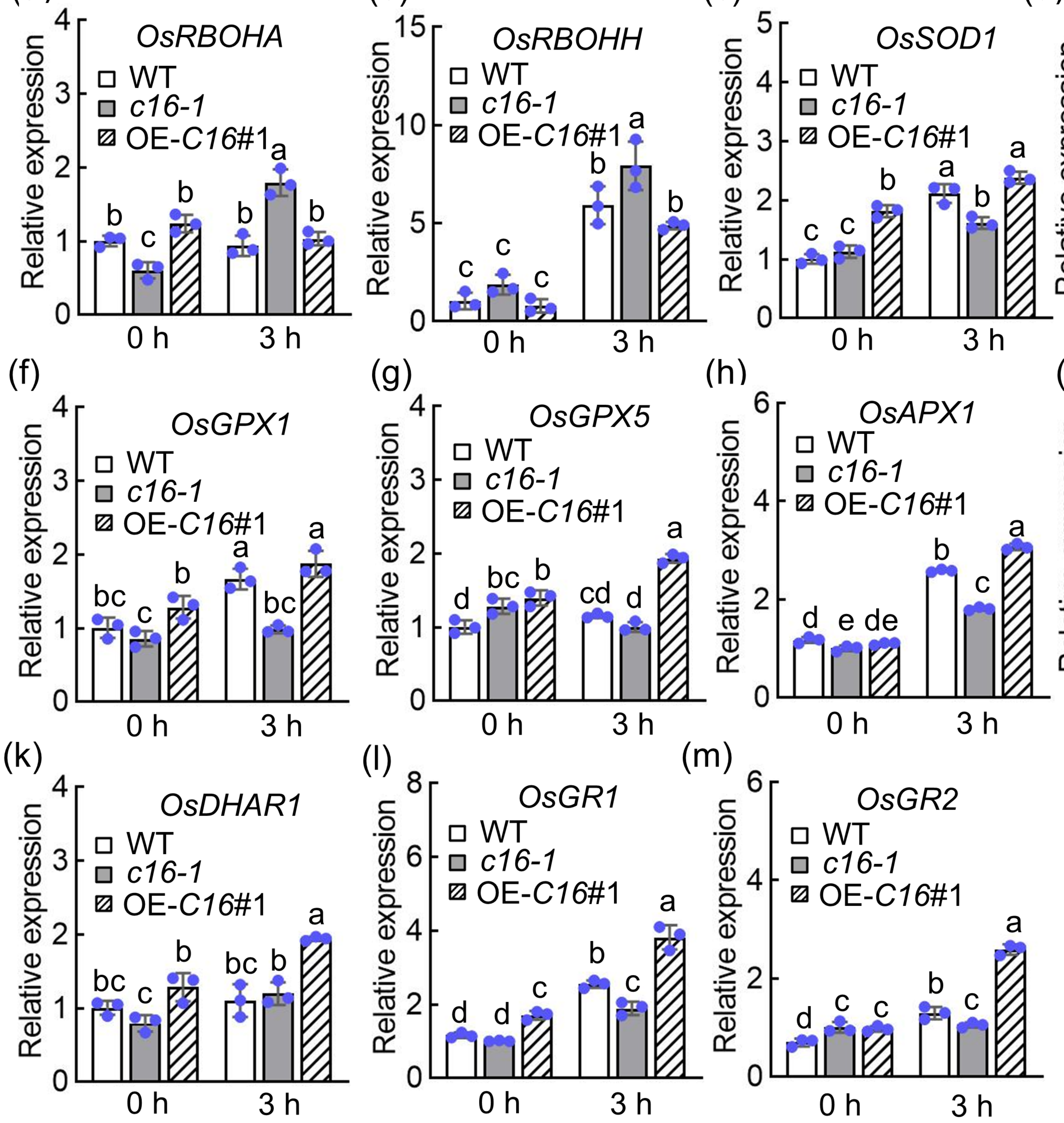

Figure S7. Expression patterns of ROS homeostasis-related genes in the *cngc16* and OE-*OsCNGC16* plants under salt stress.

Relative expression levels of ROS homeostasis-related genes in the leaf tissues of 2-week-old WT, *c16-1* and OE-*C16*#1 seedlings exposed to 130 mM NaCl for 0 h and 3 h. Transcript levels were measured by qRT-PCR and represented as relative to the level of 0 h in WT. *OsActin1* was used as the reference gene. Shown are the means ± SD from three biological replicates. Letters in indicate significant differences at $p < 0.01$ by Duncan's multiple range test via ANOVA.

Table S1. List of primers in this study.

Primer	Sequence (5'-3')	Purpose
P2YC-CDPK5-F<br>P2YC-CDPK5-R	CATTTACGAACGATAGTTAATTAAATGGGGAACACGTGCGG<br>CACTGCCACCTCCTCCACTAGTTCTCATGCTTATATTCAAG	Clone *CDPK5* gene into P2YC vector
P2YC-CDPK13-F<br>P2YC-CDPK13-R	CATTTACGAACGATAGTTAATTAAATGGGCAACGCATGCGG<br>CACTGCCACCTCCTCCACTAGTGAGTGCACCAGGTGCGTC	Clone *CDPK13* gene into P2YC vector
P2YC-R	GCTCAGGTAGTGGTTGTCGG	Sequence the P2YC vector
pBT3-SUC-CNGC16-F<br>pBT3-SUC-CNGC16-R	AATTCCTGCAGGGCCATTAATGTCAGGCGAGCTC<br>AGCTACTTACCATGGGGCCGTTAGAAGTCGTCGTC	Clone *CNGC16* gene into pBT3-SUC vector
pPR3-N-CPK5-F<br>pPR3-N-CPK5-R	TGGTATCAACGCAGAGTGGCCATTAATGGGGAACACGTGC<br>TGATATCGAATTCTCGAGAGGCCGTCATCTCATGCTTAT	Clone *CDPK5* gene into pPR3-N vector
pPR3-N-CPK13-F<br>pPR3-N-CPK13-R	TGGTATCAACGCAGAGTGGCCATTAATGGGCAACGCATGC<br>TGATATCGAATTCTCGAGAGGCCGCTAGAGTGCACCAG	Clone *CDPK13* gene into pPR3-N vector
pBT3-SUC-F<br>pBT3-SUC-R	TGGCATGCATGTGCTCTG<br>GTAAGGTGGACTCCTTCT	Sequence the pBT3-SUC vector
pPR3-N-F<br>pPR3-N-R	GTCGAAAATTCAAGACAAGG<br>AAGCGTGACATAACTAATTAC	Sequence the pPR3-N vector
CPK5-Myc-F<br>CPK5-Myc-R	TTCGAAATCGATGGATCCTAATGGGGAACACGTGCGG<br>CGATCGGGGAAATTCGAGCTCTCTCATGCTTATATTCAAG	Clone *CDPK5* gene into pCAMBIA1300 vector fusion *Myc*
CPK13-Myc-F<br>CPK13-Myc-R	TTCGAAATCGATGGATCCTA ATGGGCAACGCATGCGG<br>CGATCGGGGAAATTCGAGCTCGAGTGCACCAGGTGCGTC	Clone *CDPK13* gene into pCAMBIA1300 vector fusion *Myc*
Myc-F<br>Myc-R	CGATCGTTCAAACATTTG<br>CTATCGCGTATTAAATGT	Sequence the pCAMBIA1300 vector
CPK5-GST-F<br>CPK5-GST-R	GATCTGGTTCCGCGTGGATCCATGGGGAACACGTGCGG<br>TCAGTCAGTCACGATGAATTCTCTCATGCTTATATTCAAG	Clone *CDPK5* gene into pGEX-2T vector fusion *GST*
CPK13-GST-F<br>CPK13-GST-R	GATCTGGTTCCGCGTGGATCCATGGGCAACGCATGCGG<br>TCAGTCAGTCACGATGAATTCGAGTGCACCAGGTGCGTC	Clone *CDPK13* gene into pGEX-2T vector fusion *GST*
GST-F	ATGAGCGCGATGAAGGTGAT	Sequence the pGEX-2T vector
CNGC16-His-F<br>CNGC16-His-R	GACAGCCCAGATCTGGGTACCATGTCAGGCGAGCTCTCCA<br>ACGGAGCTCGAATTCGGATCCGAAGTCGTCGTCCTGGTTG	Clone *CNGC16* gene into pFastBacHTB vector fusion *HIS*
His-F	AGATCGATCTCGATCC	Sequence the pFastBacHTB vector
Osactin-F<br>Osactin-R	AGGAAGGCTGGAAGAGGACC<br>CGGGAAATTGTGAGGGACAT	qRT-PCR
OsHAK1-RT-F<br>OsHAK1-RT-R	CCAGTGCCAGTCACACACTT<br>CAGCTGACAGAACTGAGATGGC	qRT-PCR
OsSOS1-RT-F<br>OsSOS1-RT-R	GGAAGGGTTTGCGGTCCAA<br>GCAACCTCACTATCACCAAGAAAG	qRT-PCR
OsSOS2-RT-F<br>OsSOS2-RT-R	TTATGTTGCTCCTGAGGTGCTTA<br>CACAAGAGGGCCACCATCATT	qRT-PCR
OsNHX1-RT-F<br>OsNHX1-RT-R	TCAAGAAGCTATACATTGGAAGGCA<br>TACGGTGAGAATGCCGCTCA	qRT-PCR
OsHKT1;5-RT-F<br>OsHKT1;5-RT-R	GACAAACACCAGCAAACGGG<br>TGCCATACGCACTGATAACCTC	qRT-PCR
OsHKT2;1-RT-F<br>OsHKT2;1-RT-R	GCTTGCCTGCATCTCAGTCT<br>CATTCCCATATGCACTGATGATTTC	qRT-PCR

Primer	Sequence (5’-3’)	Purpose
OsRAB16A-RT-F<br>OsRAB16A-RT-R	TCGACGAGTACGGCAAC<br>CTCATCGGCTGGAACTG	qRT-PCR
OsNCED5-RT-F<br>OsNCED5-RT-R	TTCAAGCTCGGCGAGATGTT<br>AGGTGGAAGCAGAAGCAGTC	qRT-PCR
OsBZIP23-RT-F<br>OsBZIP23-RT-R	GCGAGGCAGGGGTCGGTGTAC<br>CCGACGGCGTGCGACTCCT	qRT-PCR
OsABI5-RT-F<br>OsABI5-RT-R	CACGATGATCAAGAGGT<br>GAGAGGATTGACGACTG	qRT-PCR
OsRBOHA-RT-F<br>OsRBOHA-RT-R	ATCACCATGCTCCAAGCTCT<br>AAATGTGTCCGGACTTTTGTC	qRT-PCR
OsRBOHH-RT-F<br>OsRBOHH-RT-R	GGATCACGGCTCAGTTGCTG<br>GCATTCTTGTCAACCATGTCAAA	qRT-PCR
OsSOD1-RT-F<br>OsSOD1-RT-R	CCATCATTGGCCGAGCTGTT<br>TGGAGTCCGATGATTCCGCAAG	qRT-PCR
OsCatA-RT-F<br>OsCatA-RT-R	TCCACCGTCATCCACGAGC<br>CCAGTACTCCTGGACATG	qRT-PCR
CatB-RT-F<br>CatB-RT-R	TACCGGTCATTTGACCCTGC<br>ATCACACTGCGACCAGTAGG	qRT-PCR
OsGPX1-RT-F<br>OsGPX1-RT-R	ATCGTTAACGTCGCATCCCA<br>TCCAAACTGATTGCACGGGA	qRT-PCR
OsGPX5-RT-F<br>OsGPX5-RT-R	ATGGCGGCTACAACTACAAGT<br>CCTTGACGGAGATGTCGTGG	qRT-PCR
OsAPX1-RT-F<br>OsAPX1-RT-R	AAGAACTACCCCGTCGTGAG<br>GGGTCTTCATCGTCCCGAAC	qRT-PCR
OsAPX3-RT-F<br>OsAPX3-RT-R	ACCGATGAAACAGAAGCATCCC<br>CGCAGCACCTTTCTTAGCATC	qRT-PCR
OsMDHAR3-RT-F<br>OsMDHAR3-RT-R	TCAAGGGAAAAGAGTCCGGC<br>GTTGTCGCCGTAGAATTGCC	qRT-PCR
OsDHAR1-RT-F<br>OsDHAR1-RT-R	TCCGCATGACAATCACGCTA<br>GGCGAGATGACCGGAGAAAT	qRT-PCR
OsGR1-RT-F<br>OsGR1-RT-R	TTCAGGCCACTTAGGGCAAC<br>GGTGCGTCTTCACCACACAT	qRT-PCR
OsGR2-RT-F<br>OsGR2-RT-R	GCTAAGGTTGCGATTTGCGA<br>TCCGCGGAAAGATGAACCAT	qRT-PCR
